# Prediction of steel plate-based damper for improving the behavior of concentrically braced frames based on RSM and ML approaches for sustainable structures

**DOI:** 10.1038/s41598-024-54845-9

**Published:** 2024-02-19

**Authors:** Kennedy C. Onyelowe, Jorge Luis Yaulema Castañeda, Ali F. Hussain Adam, Diego Ramiro Ñacato Estrella, Nakkeeran Ganasen

**Affiliations:** 1 Department of Civil Engineering, Michael Okpara University of Agriculture, Umudike, Nigeria; 2 Department of Civil Engineering, School of Engineering Technology, University of the Peloponnese, Patras, Greece; 3 Facultad de Ciencias, Escuela Superior Politecnica de Chimborazo (ESPOCH), Grupo de Investigacion LEISHPAREC, Panamericana Sur Km. 1 ½, Riobamba, 060155 Ecuador; 4 Department of Civil Engineering, College of Engineering Technologies, Club Road, Al-Qubbah, Libya; 5 Facultad de Informatica y Electronica, Escuela Superior Politecnica de Chimborazo (ESPOCH), Panamericana Sur Km. 1 ½, Riobamba, 060155 Ecuador; 6 Department of Civil Engineering, SRM Institute of Science and Technology, Kattankulathur, India

**Keywords:** Steel plate-based damper, Concentrically-braced frames, Machine learning, Response surface methodology (RSM), Stiffness, Sustainable steel structures, Civil engineering, Structural materials

## Abstract

The stiffness (K) and slenderness factor (λ) of a steel plate-based damper has been studied on the basis of elastic-inelastic-plastic buckling (EIP) modes and flexural/shear/flexural-shear failure mechanisms (FSF-S), which has been designed for the improvement of the behavior of concentrically braced frames. Steel plate-based dampers offer significant benefits in terms of mode shapes and failure mechanisms, contributing to improved dynamic performance, enhanced structural resilience, and increased safety of civil engineering structures. Their effectiveness in mitigating dynamic loads makes them a valuable tool for engineers designing structures to withstand extreme environmental conditions and seismic events. This study was undertaken by using the learning abilities of the response surface methodology (RSM), artificial neural network (ANN) and the evolutionary polynomial regression (EPR). Steel plate dampers are special structural designs used to withstand the effect of special loading conditions especially seismic effects. Its design based on the prediction of its stiffness (K) and slenderness factor (λ) cannot be overlooked in the present-day artificial intelligence technology. In this research work, thirty-three entries based on the steel plate damper geometrical properties were recorded and deployed for the intelligent forecast of the fundamental properties (λ and K). Design ratios of the steel plate damper properties were considered and models behavior was recorded. From the outcome of the model, it can be observed that even though the EPR and ANN in that order outclassed the other techniques, the RSM produced model minimization and maximization features of the desirability levels, color factor scales and 3D surface observation, which shows the real model behaviors. Overall, the EPR with R^2^ of 0.999 and 1.000 for the λ and K, respectively showed to be the decisive model but the RSM has features that can be beneficial to the structural design of the studied steel plate damper for a more robust and sustainable construction. With these performances recorded in this exercise, the techniques have shown their potential to be applied in the prediction of steel damper stiffness with optimized characteristic features to withstand structural stresses.

## Introduction

A steel plate-based damper, also known as a steel plate shear wall (SPSW), is a structural system used in concentrically braced frames (CBFs) to provide lateral resistance and dampen seismic forces^1^. It consists of vertical steel plates, commonly referred to as shear walls that are connected to the building's frame. In a concentrically braced frame, diagonal braces are used to resist lateral loads. These braces are typically made of steel and are placed in a diagonal pattern throughout the building^2^. The braces are designed to yield and dissipate energy during a seismic event, effectively reducing the forces transferred to the structure. To enhance the performance of CBFs, steel plate-based dampers are often incorporated into the system^3^. These dampers consist of steel plates that are bolted to the building's columns and beams^4^. The plates act as shear walls, providing additional stiffness and strength to the structure^1^. During an earthquake, the steel plates deform and absorb energy, reducing the seismic forces transmitted to the building. The advantages of steel plate-based dampers include: Energy dissipation: The steel plates absorb and dissipate seismic energy, reducing the forces transmitted to the structure^5^. This helps to minimize structural damage and protect the building and its occupants. Ductility: Steel plates have high ductility, allowing them to undergo significant deformation without failure. This ductility enables the dampers to effectively absorb energy and withstand strong seismic forces^6^. Space efficiency: Steel plate-based dampers provide high strength and stiffness, allowing for thinner and lighter structural elements compared to other damping systems^7^. This results in more efficient use of space within the building. Design flexibility: Steel plate-based dampers can be designed to accommodate various architectural and structural configurations^8^. They can be integrated into both new construction and retrofit projects, making them versatile and adaptable to different building types^8^. It's important to note that the design and implementation of steel plate-based dampers require careful engineering analysis. The consideration of factors such as building codes, seismic design criteria, and specific project requirements is also needed. Consulting with a qualified structural engineer is essential to ensure proper design and installation of these dampers in concentrically braced frames.

The slenderness factor of a steel plate-based damper in concentrically braced frames refers to the ratio of the effective length of the damper to its least radius of gyration^9^. The slenderness factor is an important parameter in the design of steel plate-based dampers as it influences their buckling behavior and overall performance. The slenderness factor, often denoted as λ, is calculated using the following formula: λ = (K * L)/r Where:—K is the effective length factor, which depends on the boundary conditions and end restraints of the damper^10,11^. It takes into account factors such as rotational restraints, end fixity, and bracing configuration. − L is the effective length of the damper, which is the distance between the points of lateral bracing or restraint. − r is the least radius of gyration of the damper, representing its resistance to buckling. The slenderness factor λ is used to evaluate the stability of the steel plate-based damper. If the slenderness factor exceeds a critical value, it indicates that the damper is susceptible to buckling or instability. Therefore, the design of the damper should ensure that the slenderness factor remains within acceptable limits to maintain its structural integrity^12^. The specific values for the slenderness factor and the corresponding design criteria may vary depending on the design codes and standards adopted for the project. It is crucial to consult the appropriate building codes and work with a qualified structural engineer to determine the slenderness factor requirements and ensure the proper design and performance of steel plate-based dampers in concentrically braced frames.

The stiffness of a steel plate-based damper in concentrically braced frames refers to its ability to resist deformation under applied loads. It is an important characteristic that affects the overall behavior and performance of the damper^13^. The stiffness of a steel plate-based damper depends on various factors, including the material properties of the steel plates, the dimensions and thickness of the plates, and the connection details between the plates and the building frame^14^. The stiffness is typically quantified by the elastic stiffness or the effective stiffness of the damper. The elastic stiffness of a steel plate-based damper is determined by the modulus of elasticity (E) of the steel material and the geometric properties of the damper, such as plate thickness, plate dimensions, and the number of plates used. The elastic stiffness can be calculated using established engineering formulas or through finite element analysis (FEA) techniques^15,16^. The effective stiffness of a damper takes into account the influence of energy dissipation and inelastic behavior due to yielding and deformation of the steel plates. It represents the actual stiffness exhibited by the damper under dynamic loading conditions, considering both elastic and inelastic responses. The effective stiffness is typically determined through experimental testing or advanced numerical modeling techniques^14^. The stiffness of a steel plate-based damper is a critical design parameter as it affects the distribution of forces within the structure, the level of energy dissipation, and the overall seismic response. The stiffness should be carefully selected and optimized to ensure that the damper provides the desired level of damping and structural performance^17,18^. The specific stiffness requirements and design considerations for steel plate-based dampers in concentrically braced frames may vary depending on the project's seismic design criteria, building codes, and performance objectives^19^. It is important to consult with a qualified structural engineer to determine the appropriate stiffness requirements and ensure the proper design and implementation of steel plate-based dampers in concentrically braced frames.

Concentrically braced frames (CBFs) are a common structural system used in buildings to resist lateral loads, particularly seismic forces. The behavior of CBFs under these loads can be described in terms of their stiffness, strength, resistance to lateral deformation, and energy dissipation capabilities^20^. Here are some key aspects of the structural behavior of concentrically braced frames: Stiffness: CBFs provide high stiffness in the direction of the braces, which helps to limit lateral deflections and control building deformations during seismic events^21^. The diagonal braces, typically made of steel, contribute to the overall stiffness of the frame^22,23^. The stiffness of CBFs influences the distribution of forces and displacements within the structure. Strength: CBFs are designed to resist significant lateral forces, such as those generated by earthquakes^24^. The braces in CBFs are designed to yield and dissipate energy during seismic events, limiting the forces transferred to the rest of the structure. The strength of CBFs is determined by the material properties of the braces, their size, and the connections to the building frame. Ductility: Ductility refers to the ability of a structure to undergo large deformations without sudden failure. CBFs are designed to exhibit ductile behavior, allowing the braces to yield and absorb energy during seismic events. This ductility helps to dissipate seismic forces and prevent brittle failure modes. The ductility of CBFs is achieved through appropriate material selection, design detailing, and consideration of plastic hinge formation^25^. Energy Dissipation: CBFs have inherent energy dissipation capabilities due to the yielding behavior of the braces. During seismic events, the braces undergo plastic deformation, absorbing and dissipating energy^26^. This energy dissipation helps to reduce the peak forces and protect the structure from severe damage. The energy dissipation capacity of CBFs contributes to their seismic resilience. Redundancy: CBFs often exhibit redundancy, meaning that multiple paths of load transfer exist within the system^11^. This redundancy enhances the overall structural robustness and reliability, as it allows load redistribution in case of localized brace failure or damage. P-Delta Effects: CBFs are susceptible to P-Delta effects, which refer to the second-order effects caused by the combination of axial loads and lateral displacements. These effects can lead to additional moments and forces in the structure, affecting its overall stability and behavior^4^. Proper consideration of P-Delta effects is essential in the design of CBFs. The structural behavior of CBFs is influenced by various factors, including the design parameters, brace configurations, material properties, and connections^27^. The design process involves careful analysis and consideration of these factors to ensure that CBFs perform reliably and meet the desired performance objectives under lateral loads, particularly seismic forces. Consulting with a qualified structural engineer is crucial for the proper design and implementation of concentrically braced frames.

Buckling and deformation are important considerations when designing a steel plate-based damper for concentrically braced frames (CBFs)^28^. CBFs are structural systems commonly used in seismic-resistant building design. Steel plate-based dampers, also known as yielding dampers or energy dissipating devices, are installed in CBFs to absorb and dissipate energy during seismic events^29^. These dampers consist of steel plates that yield or undergo plastic deformation under cyclic loading, thereby dissipating energy and reducing the seismic forces transmitted to the building^30,31^. However, the design of steel plate-based dampers must take into account the potential for buckling and deformation. Here's how these factors are addressed: Buckling: Buckling refers to the instability and failure of slender members under compressive loads^32^. In the case of steel plate-based dampers, buckling can occur in the plates due to the compressive forces generated during seismic loading. To prevent buckling, the plates are typically designed with adequate thickness and width-to-thickness ratios. The design may also include stiffeners, such as ribs or flanges, to increase the resistance to buckling. Finite element analysis and other computational methods are often used to evaluate the buckling resistance of the damper plates. Deformation: Deformation in steel plate-based dampers occurs primarily due to yielding and plastic deformation of the plates. The amount of deformation is directly related to the energy absorption capacity of the damper. The design of the damper considers the expected seismic forces and ensures that the plates have sufficient strength and ductility to undergo plastic deformation without failure. The plate thickness, material properties, and detailing of the connection between the damper and the surrounding structure are important factors in controlling the deformation^33^. During the design process, engineers use analytical methods, computer simulations, and experimental testing to verify the performance of steel plate-based dampers under seismic loading. These analyses help ensure that the dampers can effectively dissipate energy, withstand buckling, and undergo controlled deformation without compromising the overall structural integrity of the CBF.

In the context of steel plate-based dampers used in concentrically braced frames, the terms elastic, inelastic, and plastic buckling modes refer to different modes of structural response under axial compression. Elastic Buckling Mode: Elastic buckling refers to the sudden lateral instability that occurs when a slender member, such as a steel plate, is subjected to compressive loads^34^. In this mode, the steel plate bends and deforms in a stable manner until a critical load is reached. Beyond this load, the plate undergoes buckling, which means it deflects laterally in a nonlinear manner. In the elastic buckling mode, the steel plate retains its elastic behavior, meaning it can return to its original shape when the load is removed. Inelastic Buckling Mode: Inelastic buckling occurs when the steel plate undergoes permanent deformation due to compressive loads exceeding its yield strength. In this mode, the plate deforms plastically and exhibits a reduced stiffness compared to its initial state. The inelastic buckling mode is characterized by a combination of elastic and plastic deformations, where the plate does not return to its original shape after unloading. This mode is associated with larger compressive loads than those causing elastic buckling. Plastic Buckling Mode: Plastic buckling refers to the behavior of a steel plate subjected to compressive loads exceeding its ultimate strength. In this mode, the plate undergoes significant plastic deformation and experiences a loss of load-carrying capacity. Plastic buckling occurs when the material starts to yield and form permanent plastic strains throughout its cross-section. The plastic buckling mode is associated with the highest compressive loads and is typically a result of severe overloading or localized damage^35^. It is important to design steel plate-based dampers in concentrically braced frames to operate within the elastic or inelastic buckling range, where the deformations are recoverable and the structural integrity is maintained. Plastic buckling should be avoided as it represents a failure mode associated with permanent deformation and loss of load-carrying capacity. Design considerations, such as selecting appropriate plate thickness, material properties, and bracing configurations, are crucial to ensure the desired buckling behavior and performance of the dampers in the frame system.

Predicting stiffness and slenderness factor for a steel plate-based damper of concentrically braced frames using advanced machine learning techniques requires a comprehensive dataset containing input features and corresponding target values. Here's a general outline of the steps involved in developing such a prediction model: Data Collection: Gather data on steel plate-based dampers used in concentrically braced frames. This includes information on various design parameters, such as plate dimensions, material properties, brace properties, and geometric characteristics. Feature Engineering: Analyze the collected data and extract relevant features that could influence the stiffness and slenderness factor of the damper. This may involve geometric ratios, material properties, and other design variables^36,37^. Data Preprocessing: Prepare the dataset for training by performing necessary preprocessing steps, such as handling missing values, normalizing numerical features, and encoding categorical variables. Splitting the Dataset: Divide the dataset into two parts: a training set and a testing set^38,39^. The training set will be used to train the machine learning model, while the testing set will be used to evaluate its performance. Model Selection: Choose an appropriate machine learning algorithm that can effectively capture the relationship between the input features and the target variables. Options may include regression algorithms such as linear regression, decision trees, random forests, or more advanced techniques like neural networks. Model Training: Train the selected machine learning model using the training dataset. The model will learn the patterns and relationships within the data to make predictions on unseen examples^40^. Model Evaluation: Assess the performance of the trained model using the testing dataset. Common evaluation metrics for regression tasks include mean absolute error (MAE), mean squared error (MSE), and R-squared score. Hyperparameter Tuning: Fine-tune the model by adjusting its hyperparameters to optimize its performance^41^. This can be done using techniques like grid search, random search, or Bayesian optimization. Model Deployment: Once satisfied with the model's performance, deploy it for real-world predictions. This may involve creating an interface or integrating it into an existing system. Monitoring and Maintenance: Continuously monitor the model's performance and update it periodically to account for any changes in the underlying data or design requirements^42^. It's important to note that the specific implementation details and choice of machine learning algorithms may vary based on the characteristics of the dataset and the desired level of accuracy. Additionally, a substantial amount of accurate and representative data is crucial for training a reliable prediction model.

**Figure 1:**
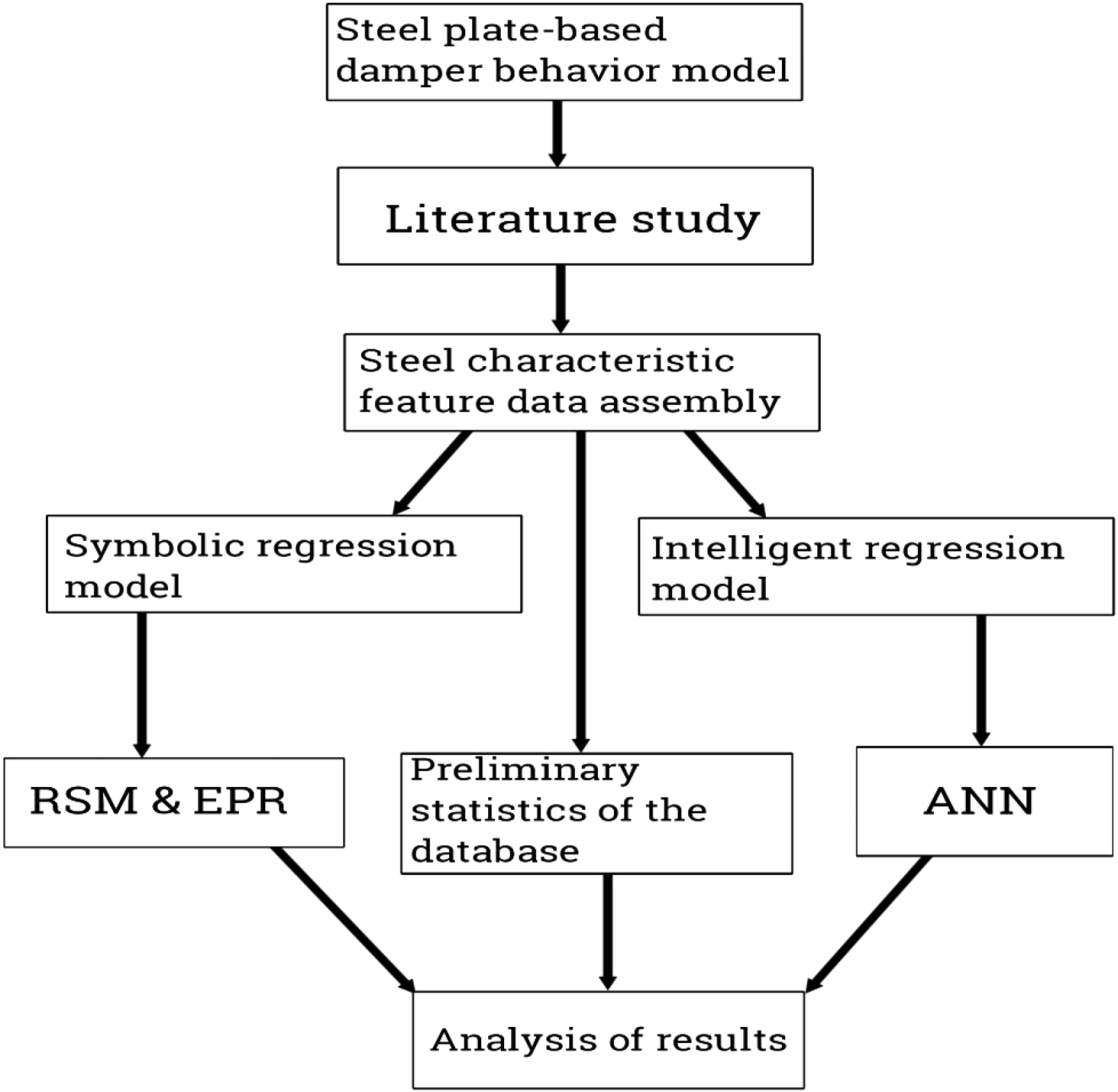
Research flowchart.

Response Surface Methodology (RSM) is a collection of statistical and mathematical techniques used for designing experiments, developing models, and optimizing processes. It is commonly used in various fields, including engineering, chemistry, and manufacturing. The basic idea behind RSM is to explore the relationship between input variables (factors) and output responses of a system^43^. By conducting a series of experiments and analyzing the data, RSM allows you to construct a mathematical model that describes the relationship between the factors and the response. This model can then be used for optimization and prediction purposes. Here are the key components and equations commonly used in RSM: Experimental design: RSM typically employs a design matrix to determine the combination of factor levels for conducting experiments. The most commonly used design is the Central Composite Design (CCD), which consists of a combination of factorial points, axial points, and center points^44^. Response function: The response function represents the relationship between the factors and the response variable. It is typically assumed to be a second-order polynomial function in RSM. The general form of the response function for a system with 'k' factors is: Y = β_0_ + β_1_X_1_ + β_2_X_2_ + … + β_i_X_i_ + β_ij_X_i_X_j_ + ɛ. In this equation, Y represents the response, X_1_, X_2_, …, X_i_ represent the factors, β_0_, β_1_, β_2_, …, β_i_, β_ij_ are the regression coefficients, X_i_X_j_ represents the interaction between factors, and ɛ represents the error term. Model fitting: The regression coefficients in the response function can be estimated using various techniques, such as the method of least squares. By fitting the model to the experimental data, the coefficients can be determined. Analysis of variance (ANOVA): ANOVA is used to assess the significance of the model terms and identify the factors that have a significant impact on the response. It helps in understanding the relative importance of the factors and their interactions^45^. Optimization: Once the model is developed and validated, RSM allows for optimization of the response. The goal is to find the factor levels that optimize the response variable. Techniques like gradient descent, response surface methodology, or numerical optimization algorithms can be used for this purpose. Validation: After optimization, the model needs to be validated to ensure its accuracy and reliability. This can be done by conducting additional experiments and comparing the predicted responses with the actual observed values. These are the fundamental elements and equations used in Response Surface Methodology. By applying these techniques, RSM helps in understanding the relationship between factors and responses, optimizing processes, and improving product or process performance. In the context of structural design of steel structures, Response Surface Methodology (RSM) can be used to develop models that relate various design factors to the response variables of interest, such as structural performance, safety, or cost^46^. These models can then be used for optimization or prediction purposes. Here is a general approach to solving an RSM model in the structural design of steel structures: Define the design factors: Identify the key design factors that impact the structural performance or behavior of the steel structure. These factors could include dimensions, material properties, connection details, or loadings. Determine the response variables: Determine the response variables that you want to optimize or predict. These variables could be aspects of structural behavior, such as strength, stiffness, deflection, or cost. Experimental design: Determine an appropriate experimental design to collect data for fitting the response surface model. This involves selecting the levels of each design factor and planning the experiments accordingly. The Central Composite Design (CCD) is a commonly used design for RSM^47^. Conduct experiments: Conduct the planned experiments according to the selected design. Each experiment involves selecting specific factor levels and collecting data on the response variables. Fit the response surface model: Use the collected data to fit a response surface model that represents the relationship between the design factors and the response variables. This can be done through regression analysis, where you estimate the coefficients of the polynomial equation that represents the response surface. Model validation: Validate the fitted response surface model by comparing predicted responses with actual observed responses from additional experiments or data. This step ensures the accuracy and reliability of the model. Optimization: Once the response surface model is validated, you can use it for optimization. Apply optimization techniques, such as gradient-based methods or numerical optimization algorithms, to find the optimal combination of design factors that maximizes or minimizes the desired response variables^48,49^. Sensitivity analysis: Perform sensitivity analysis to understand the sensitivity of the response variables to changes in the design factors. This analysis helps in identifying critical factors and understanding their influence on the structural design. Interpretation and decision-making: Analyze the results of the response surface model and optimization to interpret the findings and make informed decisions regarding the structural design of steel structures. It's important to note that the specific equations and mathematical models used in the response surface modeling will depend on the nature of the design factors, response variables, and the complexity of the structural problem at hand. The process outlined above provides a general framework for applying RSM in the structural design of steel structures, but the details and specific implementation will vary depending on the specific context and objectives of the design project. Steel plate-based dampers used in concentrically braced frames offer several sustainability benefits: Durability: Steel is a highly durable material that can withstand harsh environmental conditions and has a long lifespan. Steel plate-based dampers are designed to provide reliable and long-lasting performance, reducing the need for frequent replacements or repairs. Recyclability: Steel is one of the most recycled materials globally. At the end of their lifespan, steel plate-based dampers can be easily recycled and used to manufacture new steel products, reducing the demand for virgin steel production and conserving natural resources. Energy Efficiency: Steel plate-based dampers are designed to absorb and dissipate energy during seismic events, reducing the structural damage to buildings. By improving the seismic performance of structures, these dampers contribute to energy efficiency by reducing the need for extensive repairs, reconstruction, and associated energy consumption^50^. Seismic Resilience: Concentrically braced frames with steel plate-based dampers can enhance the seismic resilience of buildings. By dissipating seismic energy, they help protect the integrity of the structure and ensure the safety of occupants during earthquakes. This reduces the risk of structural failure, minimizes damage, and improves the overall resilience of the building. Reduced Environmental Impact: Steel plate-based dampers have a relatively low carbon footprint compared to alternative damping systems. Steel production processes have become more energy-efficient and environmentally friendly over the years, resulting in reduced greenhouse gas emissions. Additionally, the recyclability of steel reduces the demand for primary steel production, which further lowers the environmental impact. Design Flexibility: Steel plate-based dampers offer design flexibility, allowing engineers to customize dampers according to specific project requirements. This flexibility can lead to optimized designs, efficient use of materials, and reduced waste during construction. The main aim of this research work is to conduct an extensive prediction operation on the behavior of a steel plate-based damper by using the baseline regressions, response surface methodology (RSM) with an incorporation of the analysis of variance interface, the hyper-tahn activated artificial neural network (Hyper-Tahn-ANN) and the genetic algorithm activated evolutionary polynomial regression (GA-EPR). It is to be noted that the studied steel plate-based damper has been designed for the improvement of the behavior of concentrically braced frames. Conversely, previous research papers have presented the behavior mechanism of various damper systems^29–31,35^, but none presented intelligent models like it is done in this present research paper. The research flowchart is illustrated in Fig. 1.

## Methodology

**Figure 2:**
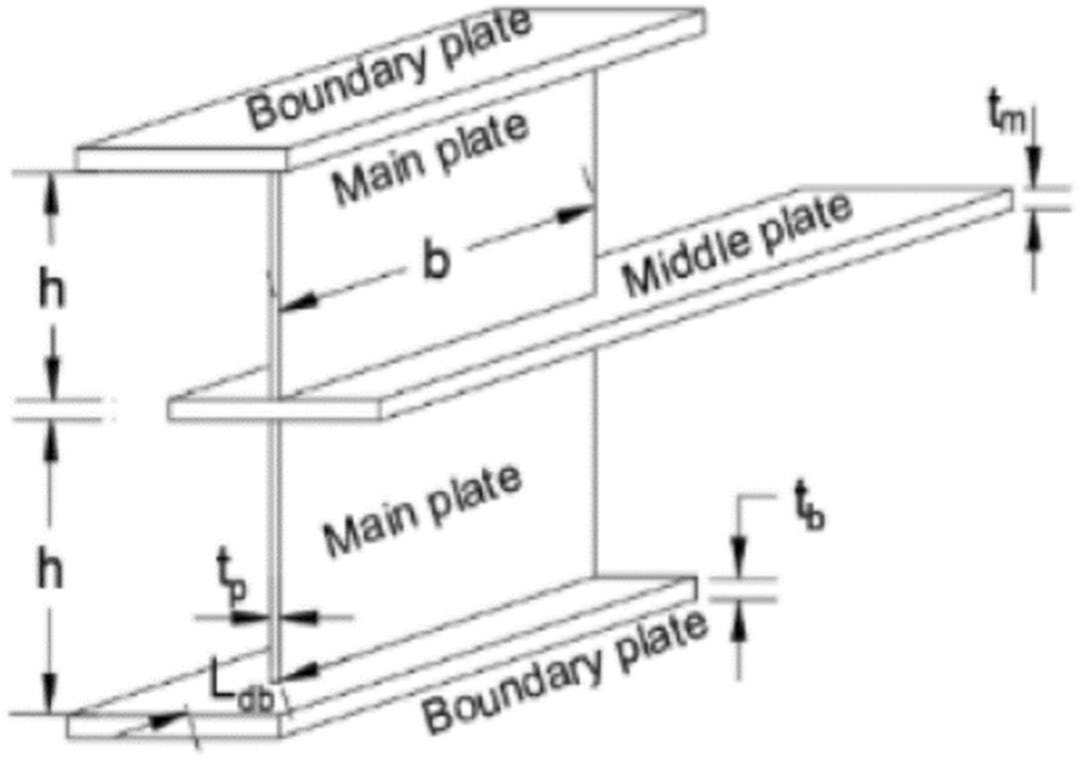
Typical steel plate-based damper showing the geometrical properties^10^.

Slenderness factor and stiffness behavior properties of a steel plate-based damper designed for the improvement of the behavior of concentrically braced frames^10^. The database was sorted and the parameters were converted to dimensionless ratios to strengthen the internal consistency of the models. Further, the impact of these parameters on the behavior of the steel damper as studied using the ANN degree of importance method. Figure 2 shows a typical steel plate-based damper showing the geometrical properties, which are measured in the 33 entries of the steel. The basic linear regression, analysis of variance (ANOVA), response surface methodology (RSM), artificial neural network (ANN) and the evolutionary polynomial regression (EPR) techniques have been extensively applied to model analyze the behavior and features of the steel plate damper for the design and construction of sustainable structures. Performance evaluation analyses were conducted based the sum of squared error (SSE), mean absolute error (MAE), mean squared error (MSE), root mean squared error (RMSE), and the R-squared indices.

### Performance analysis

Using performance parameters, the designed steel damper system's precision and error quantification have been evaluated as follows^33,38–40^;

SSE is Sum of Squares Error

1 $$SSE=\sum_{i=1}^{N}{\left({a}_{PREDICT}-\overline{a }\right)}^{2}$$

N is number of data a_PREDICT_ is actual value,$\overline{a }$ is predicted value.

MAE is the formula, which is given by Equation

2 $$MAE=\left(\frac{1}{N}\right)*\left(\sum_{i=1}^{N}\left({a}_{PREDICT}{-a}_{PREDICT}\right)\right)$$

N is the total number of trials, a_PREDICT_ is the value predicted for the jth neuron, and a_TARGET_ is the value obtained experimentally.

MSE mean squared error

3 $$MSE=\left(\frac{1}{N}\right)\sum_{i=1}^{N}{\left({a}_{PREDICT}-{\widehat{a}}_{PREDICT}\right)}^{2}$$

N is number of data a_PREDICT_ is actual value $\widehat{a}$_PREDICT_ is predicted value.

RMSE is the average deviation of a data point (targeted) from the model's predicted value (expected), expressed as the square root of the mean square error. A lower RMSE Equation indicates a better performing model

4 $$RMSE=\sqrt{\left(\frac{1}{N}\right)*\left(\sum_{i=1}^{N}{\left({a}_{PREDICT}{-a}_{PREDICT}\right)}^{2}\right) }$$

The initial parameter is the R^2^ coefficient, which represents the absolute proportion of a variable's variance.

5 $${R}^{2}=1-\left(\frac{\sum_{i=1}^{N}{\left({a}_{PREDICT}-{a}_{TARGET}\right)}^{2}}{{\sum }_{i=1}^{N}{\left({a}_{PREDICT}\right)}^{2}}\right)$$

## Results and analyses

### Basic statistical analysis

**Figure 3:**
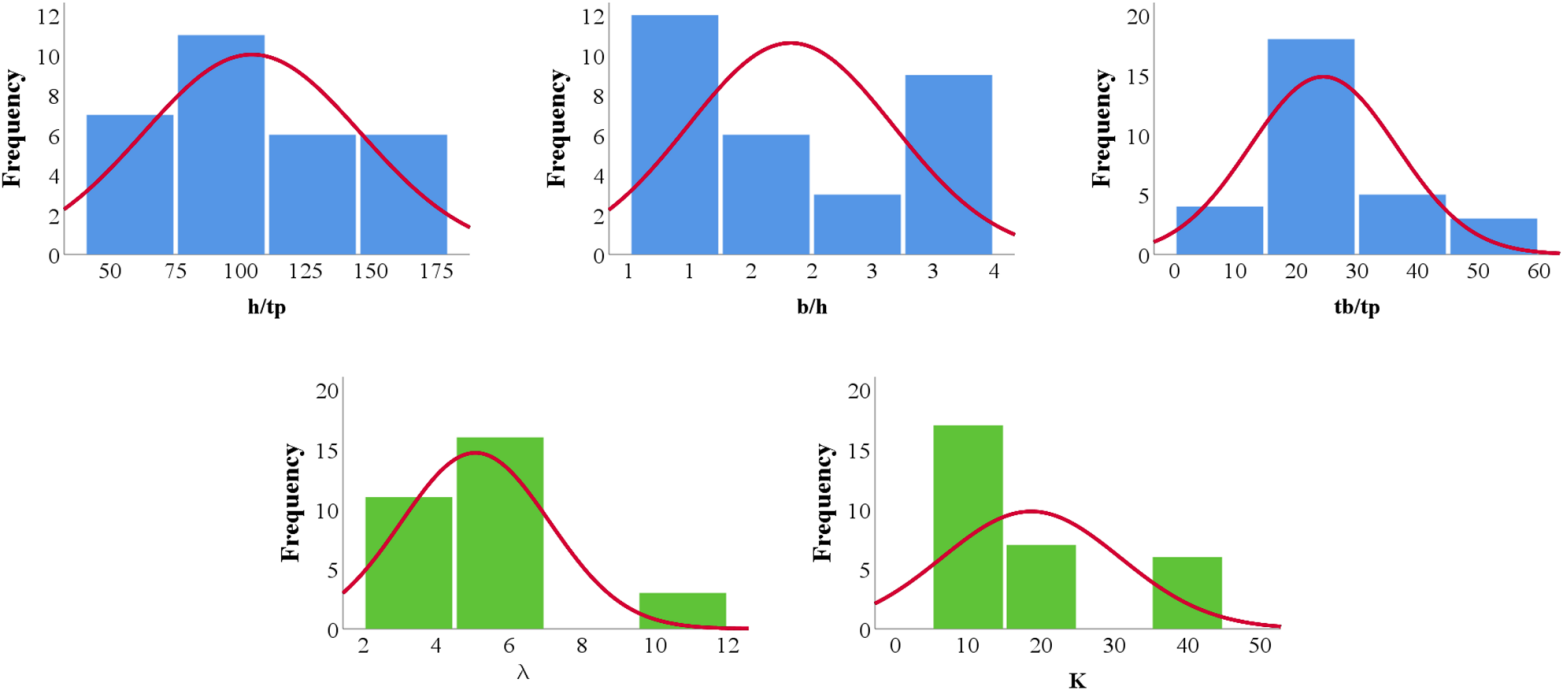
Histogram of the steel damper collected database based on properties ratios.

The general database was analyzed without considering the ratios of the damper properties as presented in Table 1. This presents the mean, standard error, median, mode, standard deviation, variance, kurtosis, skewness, range, minimum, maximum, and confidence level of the collected data for the studied steel damper. The Pearson correlation data is presented in Table 2, which shows the internal consistency of the collected data with respect to the studied parameters and characteristic features of the steel damper. The linear closed-form equations produced from the linear regression are presented in Eqs. (6), (7), (8), (9) and (10). Table 3 shows the training and validation database based on damper properties ratios and considering the failure modes and mechanics of the failures under loading. Tables 4 and 5 show the further statistical analysis of the collected database based on the training and validation regimes. Figure 3 shows the data histogram behavior with respect to the outputs.

6 $${1}.{12 }\surd {\text{k}} = {15}.{2443 } - 0.0{34}0{183 }*{\text{ b }} - 0.000{1}0{4727 }*{\text{ h }} - 0.{149857 }*{\text{ tp }} - 0.00981472 \, *{\text{ tb}}$$

7 $${1}.{4 }\surd {\text{k}} = { 19}.0{427 } - \, 0.0{42465 }*{\text{ b }} - \, 0.000{1}0{74}0{8 }*{\text{ h }} - \, 0.{191929 }*{\text{ tp }} - \, 0.0{122553 }*{\text{ tb}}$$

8 $$\lambda = { 2}.{39461 } + \, 0.0{149443 }*{\text{ b }} + \, 0.0{1}0{5237 }*{\text{ h }} - { 1}.{47}0{81 }*{\text{ tp }} - \, 0.0{2}00{139 }*{\text{ tb}}$$

9 $${\text{Lbr}} = \, - {1}.{936}0{3 } + \, 0.0{12877 }*{\text{ b }} - \, 0.00{748684 }*{\text{ h }} - \, 0.{116521 }*{\text{ tp }} + \, 0.0{123}0{92 }*{\text{ tb}}$$

10 $${\text{K}} = { 4}.{21351 } - 0.00{6983 }*{\text{ b }} - \, 0.000{15}0{795 }*{\text{ h }} - \, 0.0{272768 }*{\text{ tp }} - \, 0.00{21}0{845 }*{\text{ tb}}$$

**Table 1:** General statistical analysis of collected database.

	b (mm)	h (mm)	tp (mm)	tb (mm)	1.12 √k	1.4 √k	λ	Lbr (m)	K
Mean	292.73	186.36	1.94	41.82	4.57	5.71	5.05	0.73	2.00
St. Error	4.07	15.16	0.14	1.47	0.24	0.30	0.34	0.24	0.05
Median	300.00	170.00	2.00	40.00	3.89	4.86	5.20	0.00	1.86
Mode	300.00	100.00	2.00	50.00	7.20	9.00	5.20	0.00	2.54
St. deviation	23.35	87.06	0.83	8.46	1.38	1.73	1.95	1.40	0.29
Variance	545.45	7580.11	0.68	71.59	1.91	2.98	3.80	1.95	0.08
Kurtosis	7.34	− 1.03	1.64	− 1.52	− 0.07	− 0.07	3.55	1.40	− 0.25
Skewness	− 2.98	0.59	1.15	− 0.37	1.14	1.15	1.71	1.69	1.03
Range	80.00	250.00	3.00	20.00	4.05	5.06	8.09	4.00	0.86
Minimum	220.00	100.00	1.00	30.00	3.15	3.94	2.30	0.00	1.68
Maximum	300.00	350.00	4.00	50.00	7.20	9.00	10.39	4.00	2.54
Sum	9660.00	6150.00	63.90	1380.00	150.68	188.36	166.50	24.00	66.01
Count	33.00	33.00	33.00	33.00	33.00	33.00	33.00	33.00	33.00
Confidence level (95.0%)	8.28	30.87	0.29	3.00	0.49	0.61	0.69	0.50	0.10

**Table 2:**
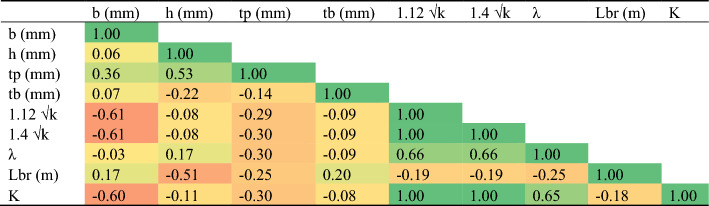
General Pearson correlation matrix.

**Table 3:** Training and validation database based on damper properties ratios.

h/tp	b/h	tb/tp	λ	K	Mode	Mech
Training set						
83.33	3.00	41.67	2.30	16.00	I	S
85.00	1.76	15.00	5.20	21.39	I	S–F
65.00	1.15	10.00	2.60	10.68	P	S
170.00	1.29	40.00	5.20	41.33	P	S–F
50.00	3.00	20.00	3.81	12.06	P	F
125.00	1.20	25.00	10.39	41.33	E	S–F
120.00	1.00	12.00	4.16	9.32	I	S
50.00	3.00	15.00	3.81	12.06	P	F
170.00	1.29	30.00	5.20	41.33	P	S–F
125.00	1.20	20.00	10.39	41.33	E	S–F
100.00	3.00	30.00	5.20	11.09	E	F
75.00	2.00	20.00	5.20	17.84	I	F
75.00	2.00	25.00	5.20	17.84	I	F
85.00	1.76	20.00	5.20	21.39	I	S–F
175.00	0.86	20.00	5.20	7.91	E	S
170.00	1.29	50.00	5.20	41.33	P	S–F
120.00	1.00	20.00	4.16	9.32	I	S
50.00	3.00	25.00	3.81	12.06	P	S
50.00	3.00	25.00	3.81	12.06	P	F
65.00	1.15	7.50	2.60	10.68	P	S
75.00	2.00	15.00	5.20	17.84	I	F
175.00	0.86	25.00	5.20	7.91	E	S
Validation set						
125.00	1.20	15.00	10.39	41.33	E	S–F
175.00	0.86	15.00	5.20	7.91	E	S
85.00	1.76	25.00	5.20	21.39	I	S–F
65.00	1.15	12.50	2.60	10.68	P	S
100.00	3.00	40.00	5.20	11.09	E	F
120.00	1.00	16.00	4.16	9.32	I	S
100.00	3.00	50.00	5.20	11.09	E	S
100.00	3.00	50.00	5.20	11.09	E	F

*E* elastic buckling, *I* inelastic buckling, *P* plastic buckling, *F* flexural failure, *S* shear failure, and *F* flexural-shear failure.

**Table 4:** The statistical analysis based on the training and validation database.

	h/tp	b/h	tb/tp	λ	K
Training set					
Max	175	3.00	50.0	10.39	41.3
Min	50	0.86	7.5	2.30	7.9
Avg	103	1.81	23.2	4.96	19.7
SD	44	0.80	10.1	1.96	12.3
Var	0.43	0.44	0.44	0.40	0.62
Validation set					
Max	175	3.00	50.0	10.39	41.3
Min	65	0.86	12.5	2.60	7.9
Avg	109	1.87	27.9	5.39	15.5
SD	31	0.91	15.2	2.08	10.5
Var	0.28	0.48	0.54	0.38	0.68

**Table 5:**
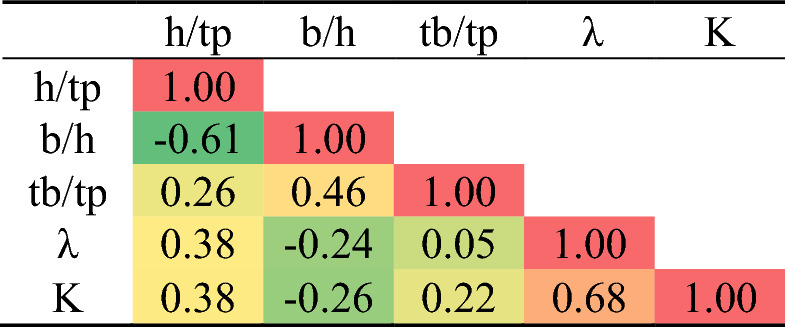
Pearson correlation of database based on damper properties ratios.

### Response surface methodology analyses

#### Steel damper stiffness (K)

The maximum model order was set to quadratic for process factors. The selected model on the Model tab may be the design model or lower in order. The fit summary calculation in Tables 6, 7 and 8 was ended prematurely based on options set on the Transform tab. Select the highest order polynomial where the additional terms are significant and the model is not aliased. It was focused on the model maximizing the Adjusted R^2^ and the Predicted R^2^.

**Table 6:** **K** fit summary.

Source	Sequential p-value	Lack of fit p-value	Adjusted R^2^	Predicted R^2^	
Linear	0.0097		0.2796	0.1963	Suggested
2FI	0.3685		0.2921	0.0979	Aliased

**Table 7:** K sequential model sum of squares [Type I].

Source	Sum of squares	df	Mean square	F-value	p-value	
Mean vs. total	131.88	1	131.88			
Linear vs. mean	0.9820	4	0.2455	4.10	0.0097	Suggested
2FI vs. linear	0.2643	4	0.0661	1.12	0.3685	Aliased
Residual	1.41	24	0.0588			
Total	134.54	33	4.08

**Table 8:** RSM model summary statistics.

Source	Std. dev	R^2^	Adjusted R^2^	Predicted R^2^	PRESS	
Linear	0.2446	0.3696	0.2796	0.1963	2.14	Suggested
2FI	0.2424	0.4691	0.2921	0.0979	2.40	Aliased

**Figure 4:**
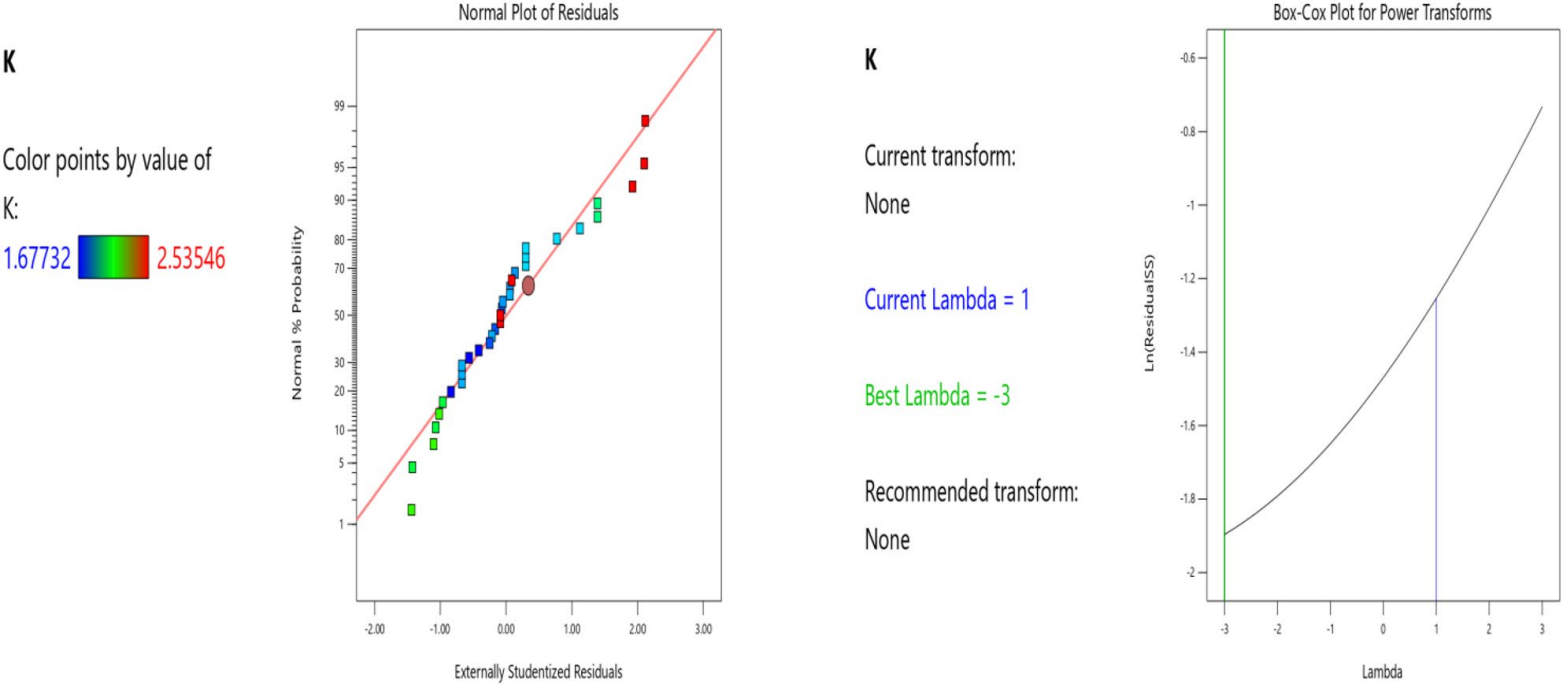
The normal plots of residuals and box-Cox power transform of K model.

**Figure 5:**
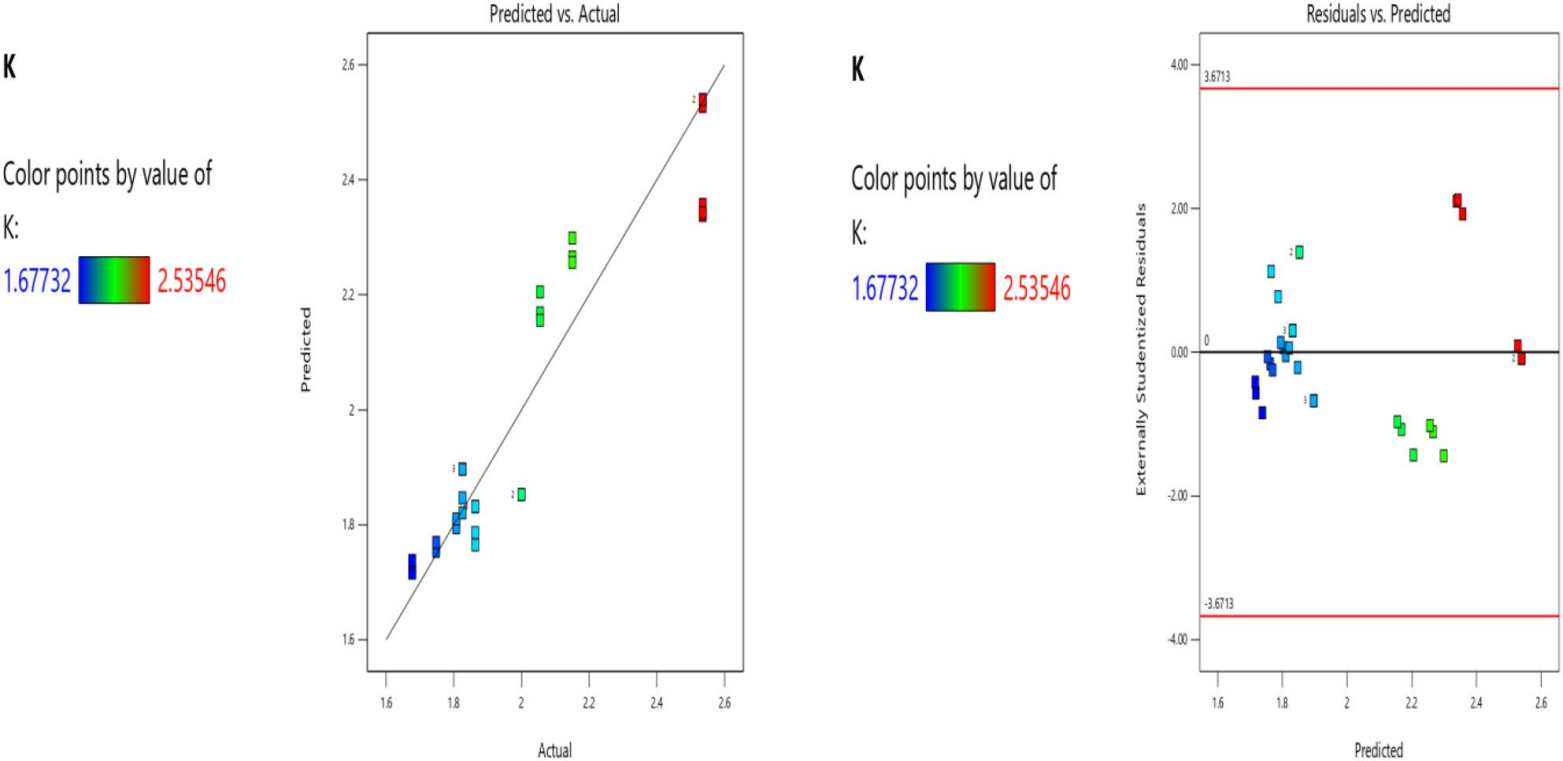
The color points for the predicted/actual and residual/predicted values.

**Figure 6:**
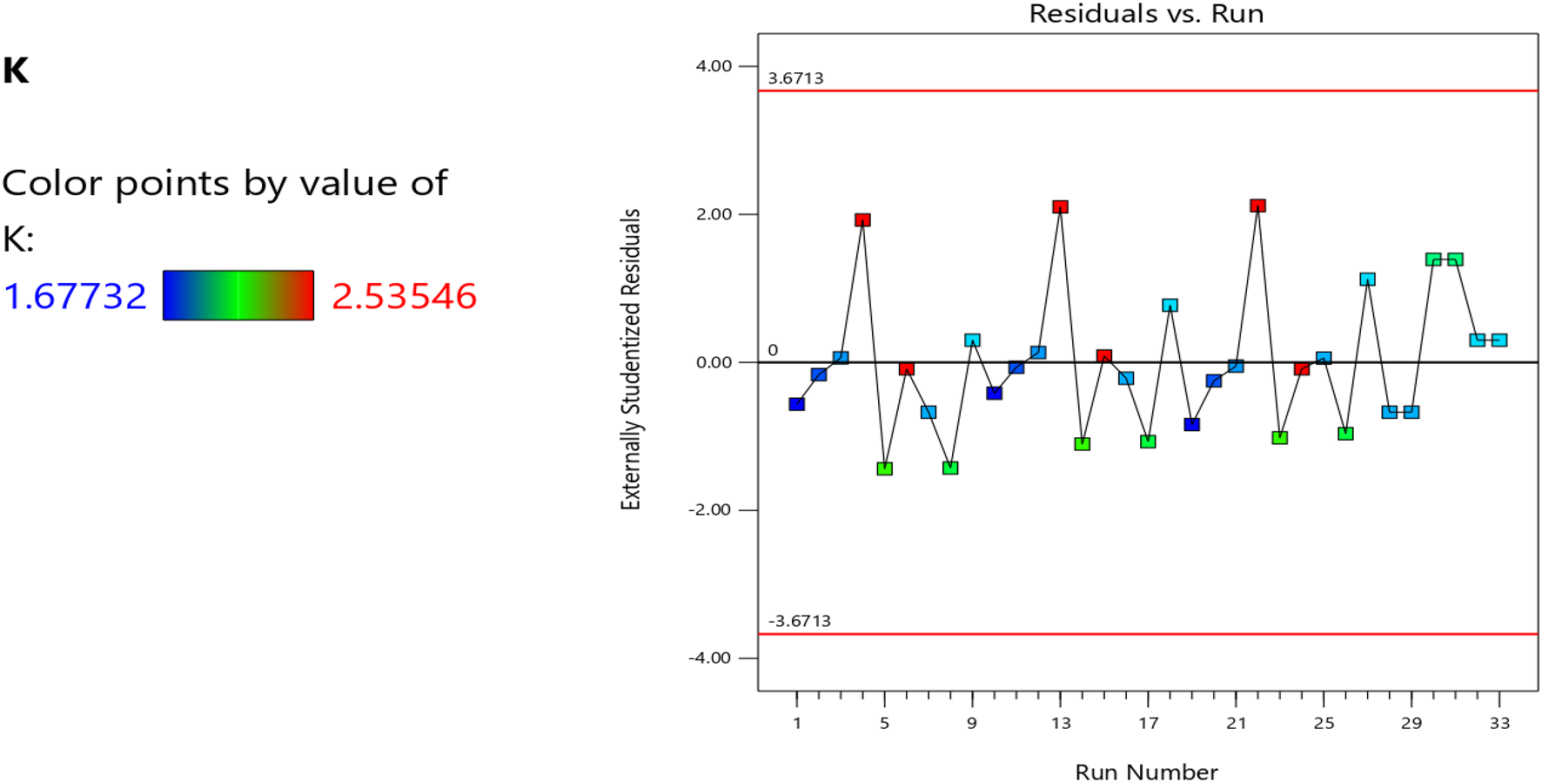
The color points for the residual/run of the RSM K model.

**Figure 7:**
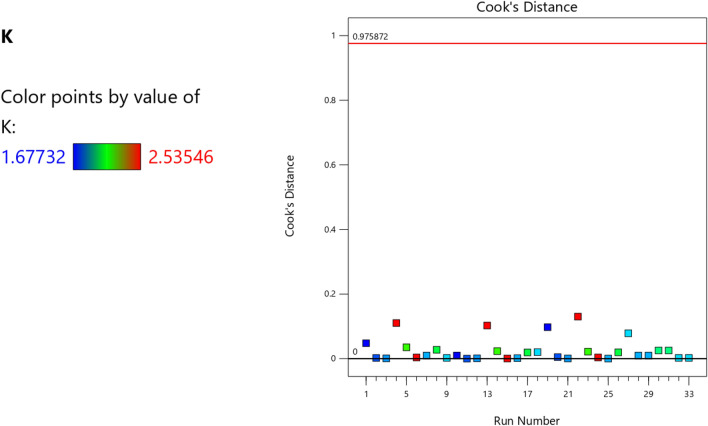
The color points for the Cook’s distance of the RSM K model.

**Figure 8:**
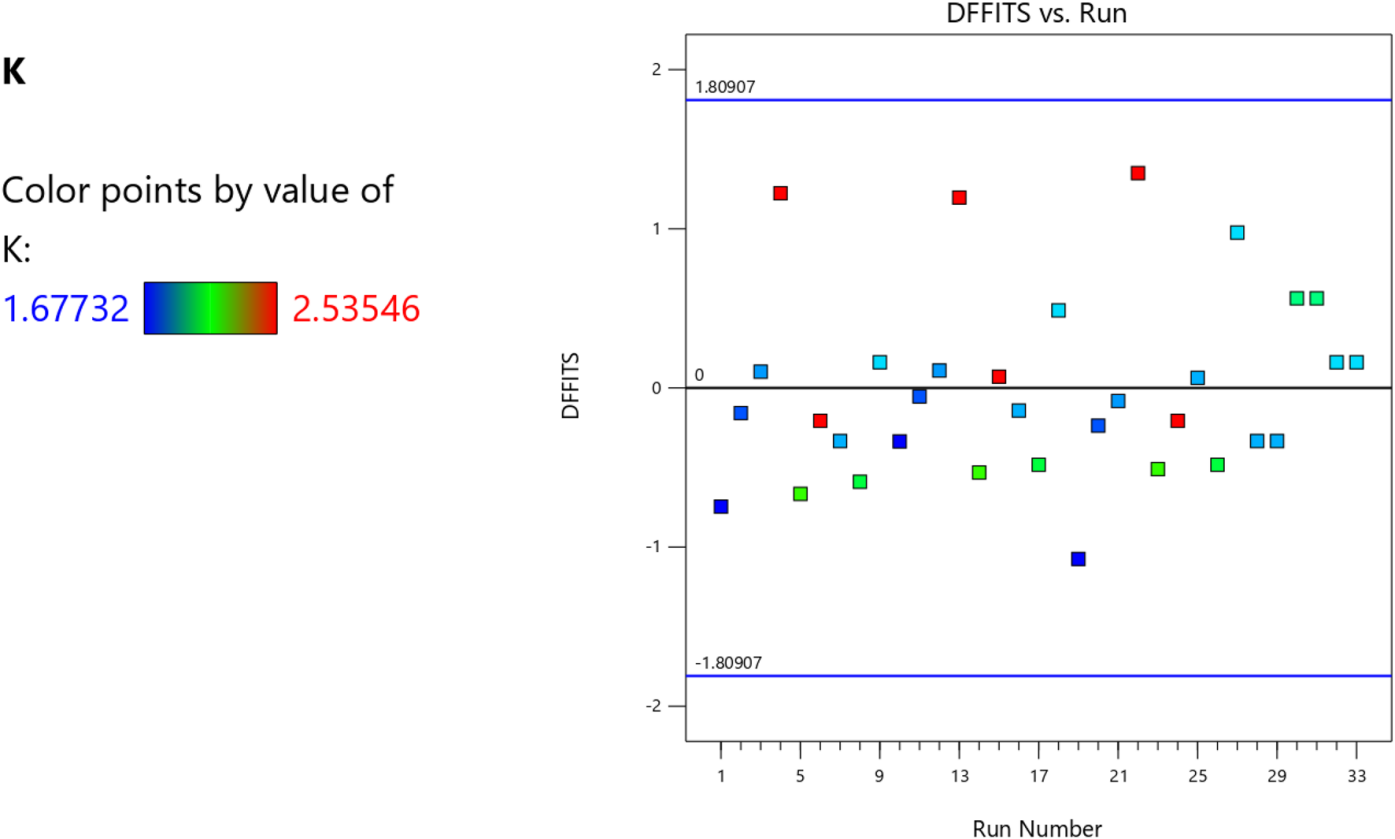
The color points for the degree of fitness of the RSM K model.

Factor coding is Actual**.** Sum of squares is Type III—Partial. The Model F-value of 15.86 implies the model is significant. There is only a 0.01% chance that an F-value this large could occur due to noise. P-values less than 0.0500 indicate model terms are significant. In this case B, BC, B2, C2 are significant model terms. Values greater than 0.1000 indicate the model terms are not significant. If there are many insignificant model terms (not counting those required to support hierarchy), model reduction may improve your model. The Predicted R^2^ of 0.8055 is in reasonable agreement with the Adjusted R^2^ of 0.8362; i.e., the difference is less than 0.2. Adeq Precision measures the signal to noise ratio. A ratio greater than 4 is desirable. Your ratio of 11.712 indicates an adequate signal. This model can be used to navigate the design space. The coefficient estimate represents the expected change in response per unit change in factor value when all remaining factors are held constant. The intercept in an orthogonal design is the overall average response of all the runs. The coefficients are adjustments around that average based on the factor settings. When the factors are orthogonal the VIFs are 1; VIFs greater than 1 indicate multi-colinearity, the higher the VIF the more severe the correlation of factors. As a rough rule, VIFs less than 10 are tolerable. The deployed model properties are presented in Tables 9, 10 and 11. The equation in terms of actual factors can be used to make predictions about the response for given levels of each factor. Here, the levels should be specified in the original units for each factor. This equation (Eq. (11)) should not be used to determine the relative impact of each factor because the coefficients are scaled to accommodate the units of each factor and the intercept is not at the center of the design space. Figures 4, 5, 6, 7 and 8 presents the ANOVA-RSM interface which modeled and optimizes the stiffness (K) of the steel damper. Overall, previous research papers have presented the behavior mechanism of various damper systems^29–31,35^, but none presented intelligent models like it is done in this present research paper.

**Table 9:** K ANOVA for Quadratic model (Aliased).

Source	Sum of squares	df	Mean square	F-value	p-value	
Model	2.37	11	0.2156	15.86	< 0.0001	Significant
A-b (mm)	0.0001	1	0.0001	0.0080	0.9297	
B-h (mm)	0.7204	1	0.7204	52.99	< 0.0001	
C-tp (mm)	0.0044	1	0.0044	0.3243	0.5751	
D-tb (mm)	0.0010	1	0.0010	0.0707	0.7929	
AB	0.0000	0				
AC	0.0000	0				
AD	0.0010	1	0.0010	0.0731	0.7895	
BC	0.1860	1	0.1860	13.68	0.0013	
BD	0.0034	1	0.0034	0.2466	0.6246	
CD	0.0002	1	0.0002	0.0127	0.9115	
A^2^	0.0000	0				
B^2^	1.07	1	1.07	78.39	< 0.0001	
C^2^	0.1036	1	0.1036	7.62	0.0117	
D^2^	0.0009	1	0.0009	0.0652	0.8009	
Residual	0.2855	21	0.0136			
Lack of fit	0.2855	16	0.0178			
Pure error	0.0000	5	0.0000			
Cor total	2.66	32

**Table 10:** Fit statistics.

Std. dev	0.1166	R^2^	0.8925
Mean	2.00	Adjusted R^2^	0.8362
C.V. %	5.83	Predicted R^2^	0.8055
		Adeq precision	11.7122

**Table 11:** Coefficients in terms of actual factors.

Factor	Coefficient estimate	df	Standard error	95% CI low	95% CI high	VIF
Intercept	− 0.2217	1	1.63	− 3.61	3.17	
A-b (mm)	0.0005	1	0.0051	− 0.0102	0.0111	33.60
B-h (mm)	0.0280	1	0.0038	0.0200	0.0360	263.39
C-tp (mm)	− 0.1371	1	0.2407	− 0.6376	0.3635	93.30
D-tb (mm)	− 0.0132	1	0.0497	− 0.1165	0.0901	415.67
AB	ALIASED					
AC	ALIASED					
AD	0.0000	1	0.0001	− 0.0002	0.0003	245.52
BC	− 0.0049	1	0.0013	− 0.0076	− 0.0021	370.18
BD	− 0.0000	1	0.0000	− 0.0001	0.0001	39.97
CD	− 0.0004	1	0.0040	− 0.0087	0.0078	48.96
A^2^	ALIASED					
B^2^	− 0.0000	1	4.470E − 06	− 0.0000	− 0.0000	64.74
C^2^	0.1939	1	0.0702	0.0478	0.3399	191.01
D^2^	0.0001	1	0.0005	− 0.0009	0.0011	240.85

11 $$K=-0.000040{h}^{2}+0.193867{tp}^{2}+0.000119{tb}^{2}+0.000457 b+0.027969 h-0.137062 tp-0.013207 tb+0.000032 b*tb-0.004871 h*tp-0.000017 h*tb-0.000446 tp*tb-0.221667$$

**Figure 9:**
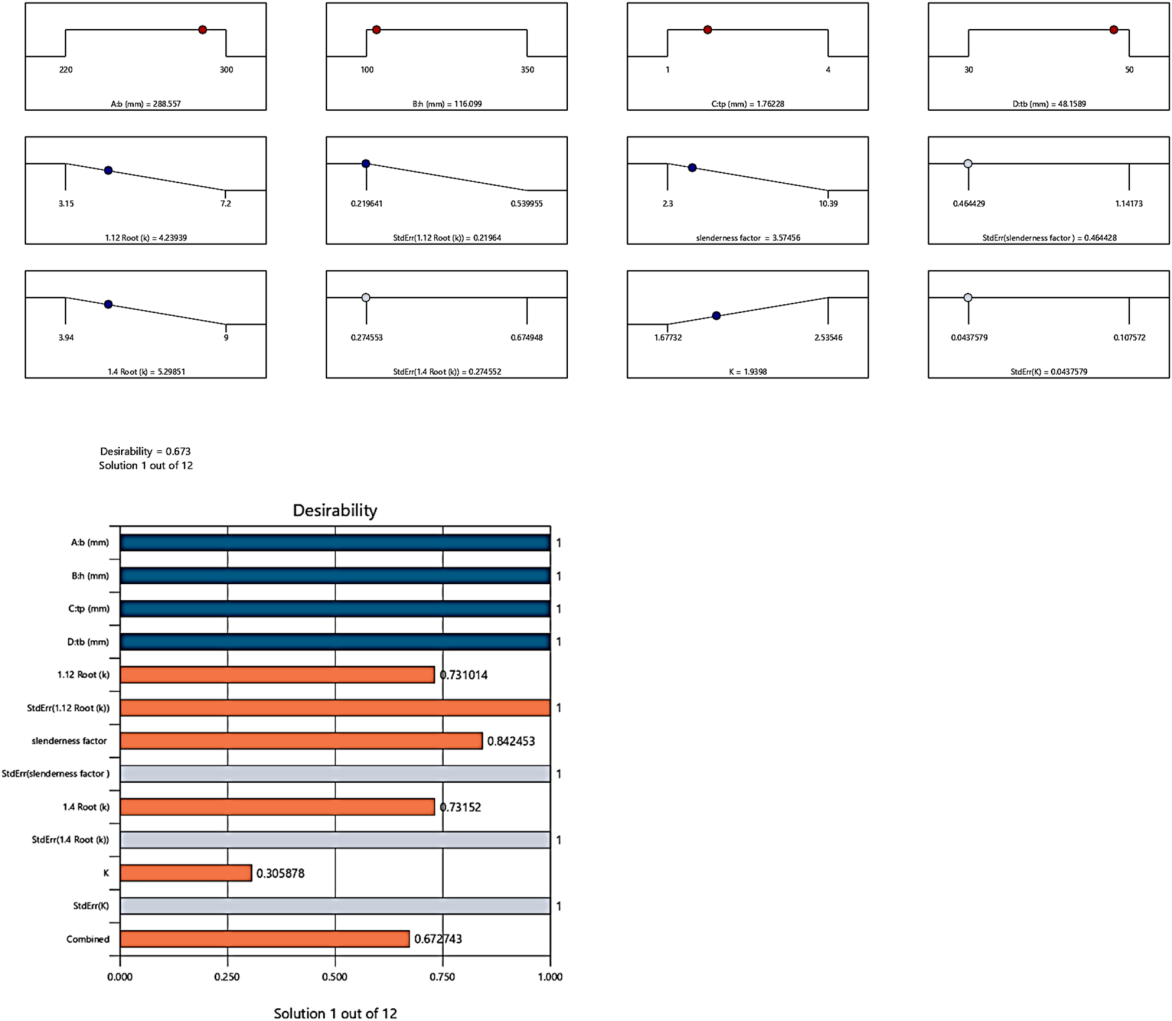
The desirability level of the RSM K maximization model.

**Figure 10:**
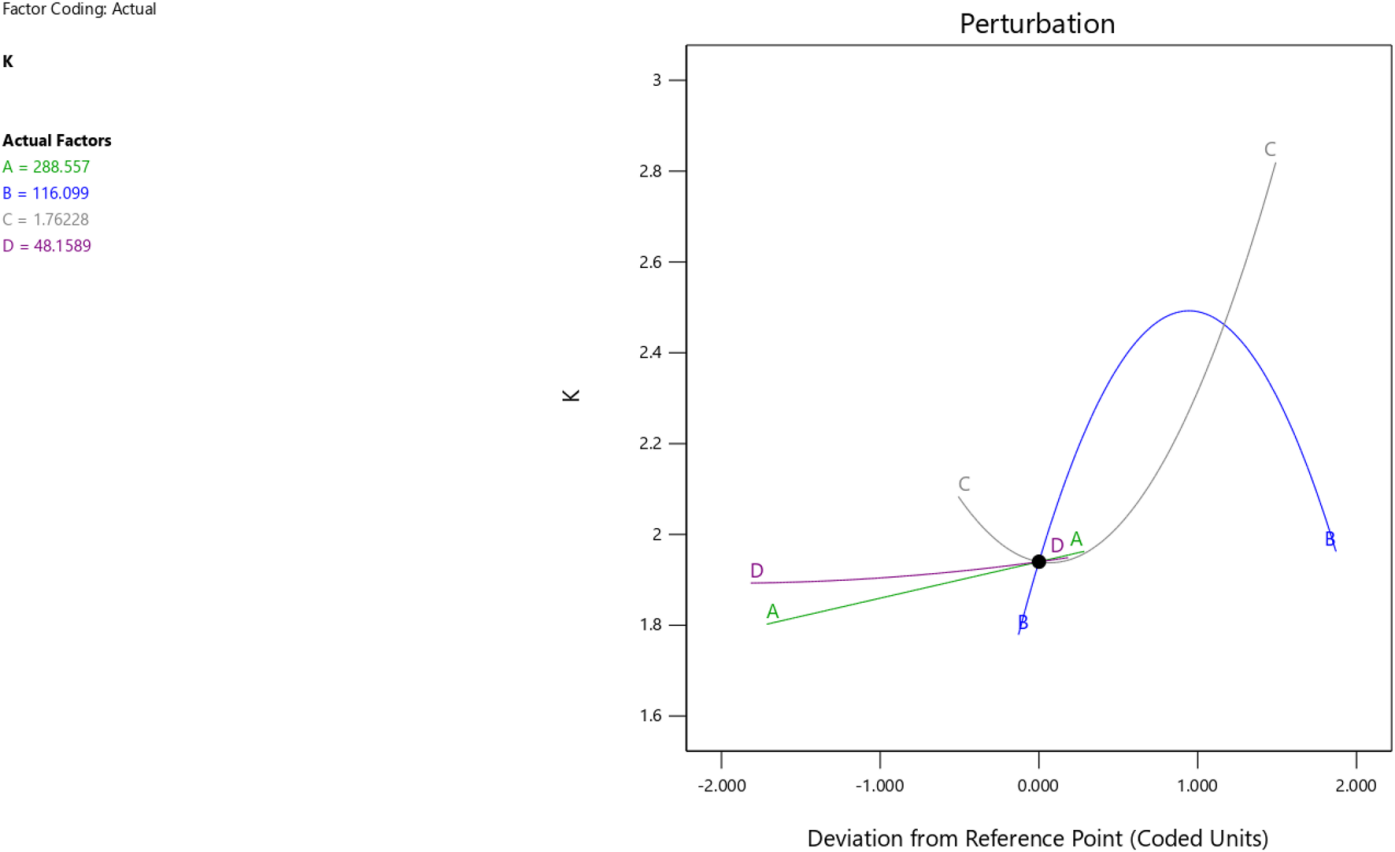
The factor coding for the perturbation of the RSM K maximization model.

**Figure 11:**
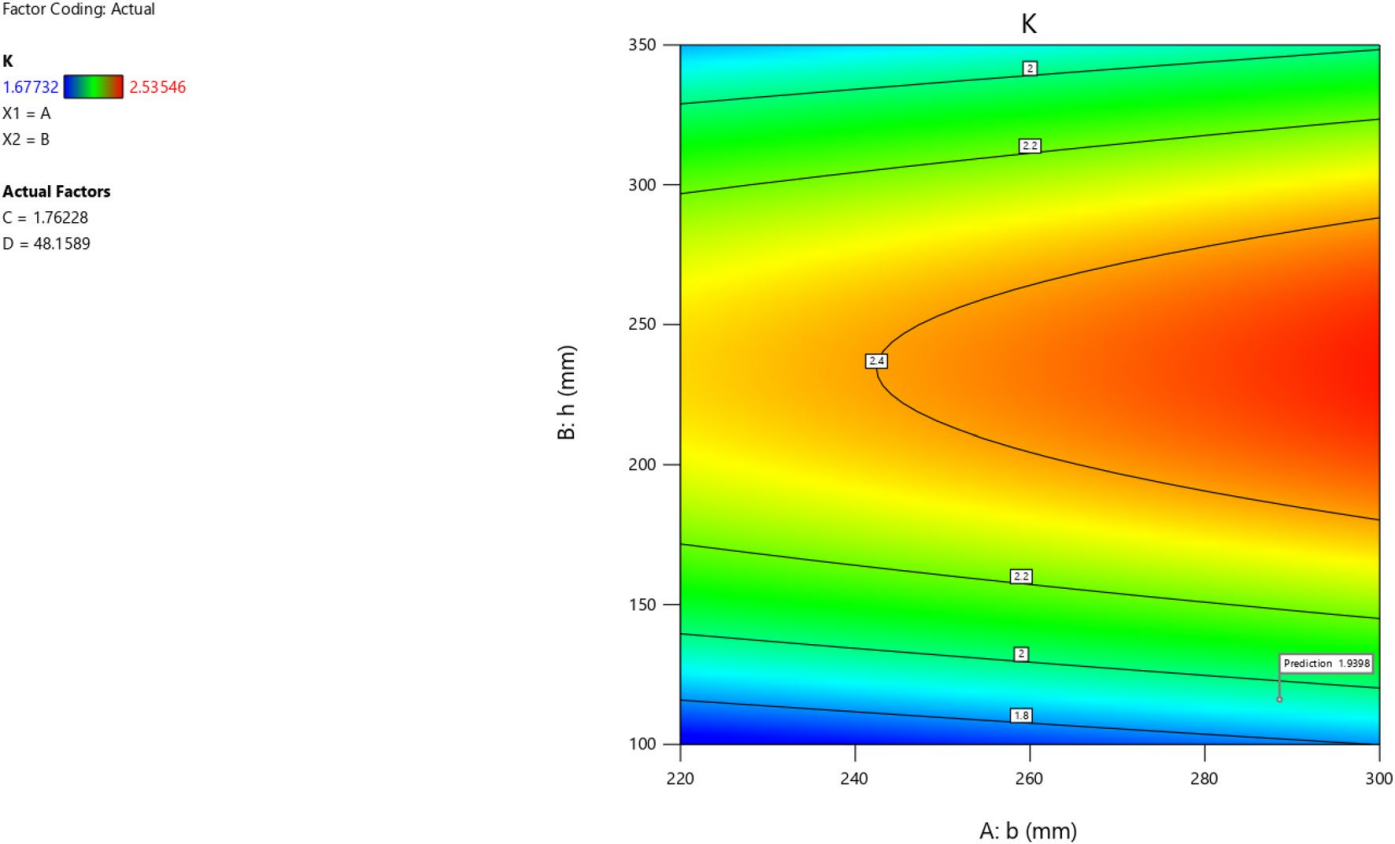
The color coding for the factor coding of the RSM K maximization model.

**Figure 12:**
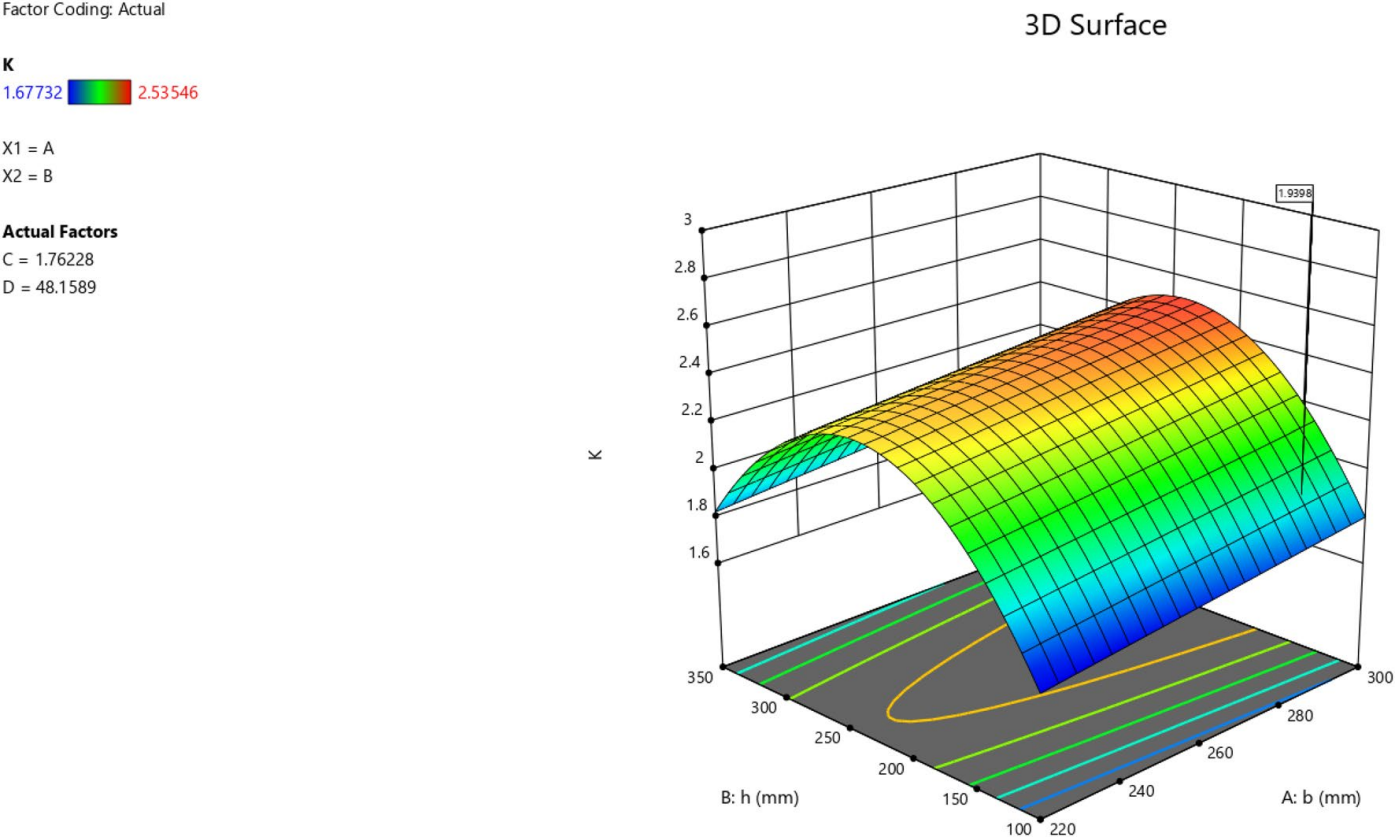
The color coding for the 3D surface of the RSM K maximization model.

**Figure 13:**
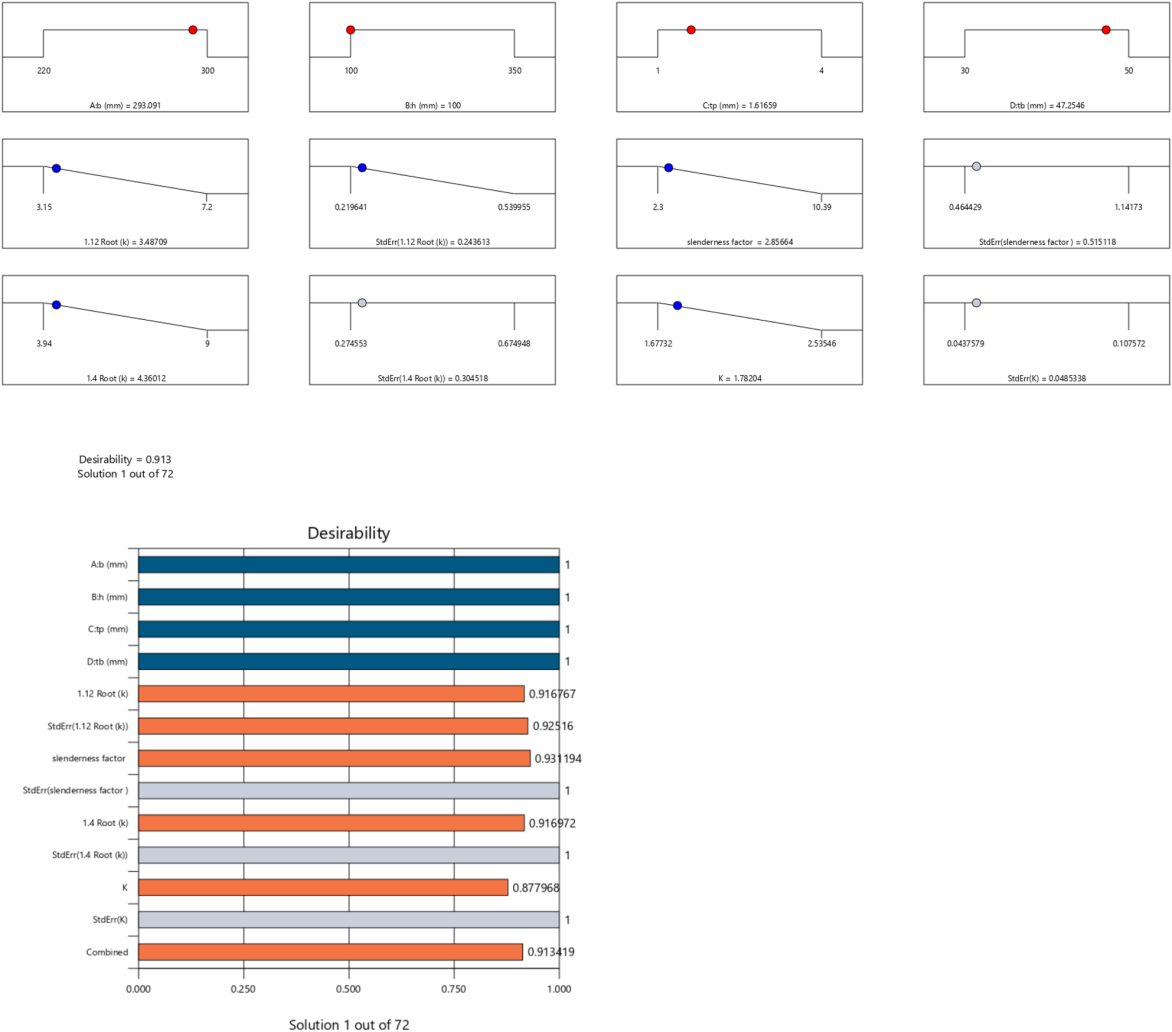
The desirability level of the RSM K minimization model.

**Figure 14:**
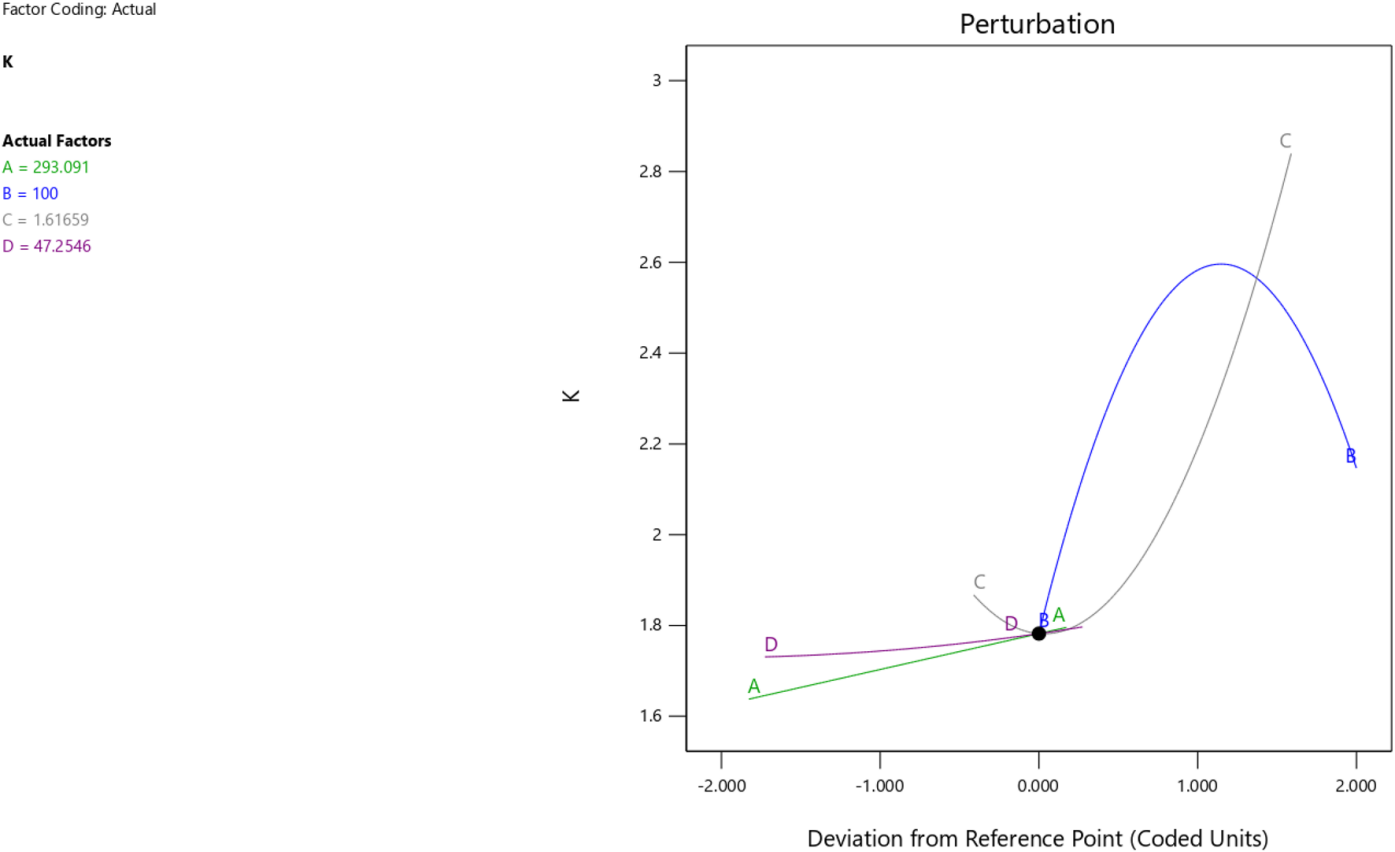
The factor coding for the perturbation of the RSM K maximization model.

**Figure 15:**
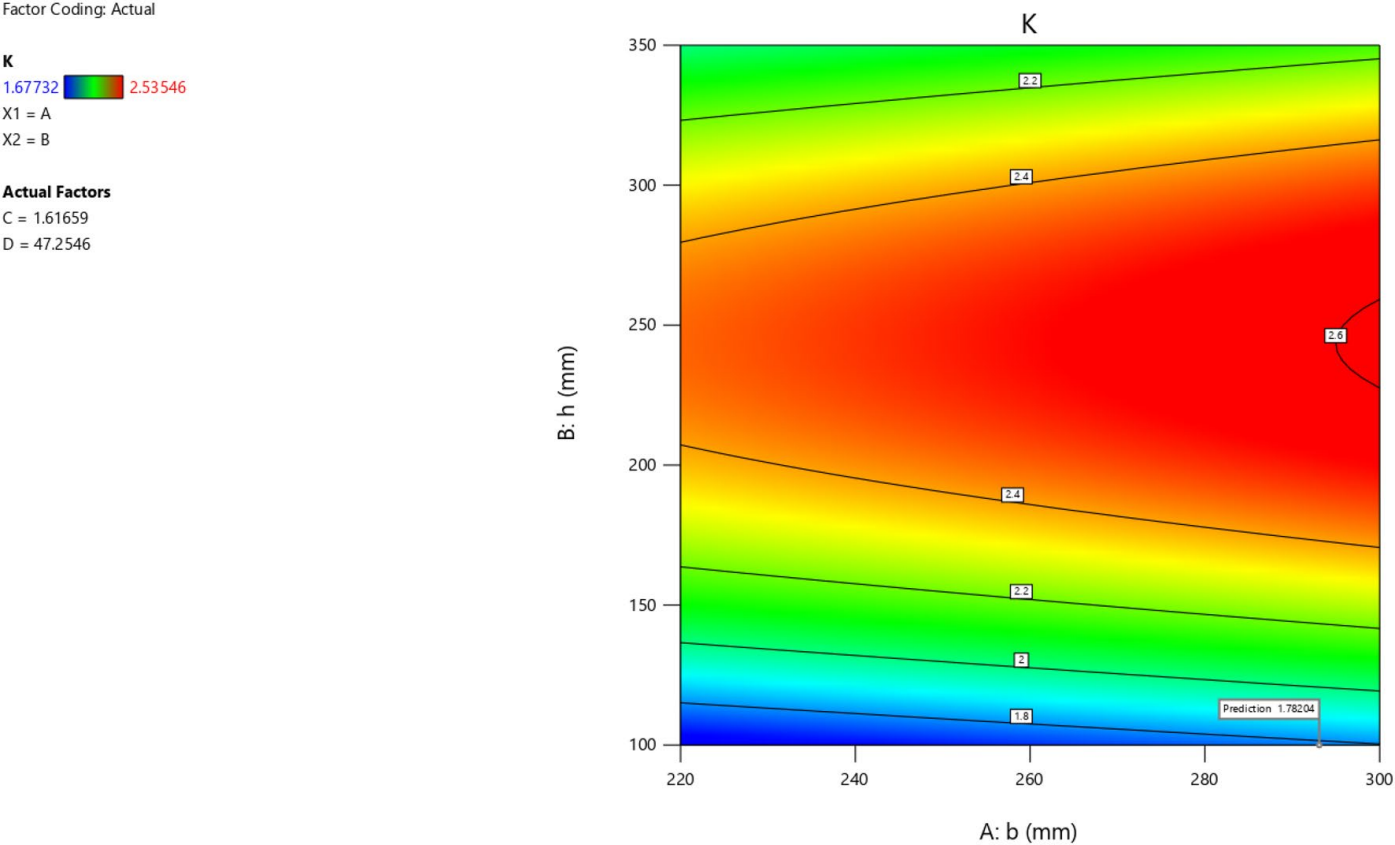
The color coding for the factor coding of the RSM K minimization model.

**Figure 16:**
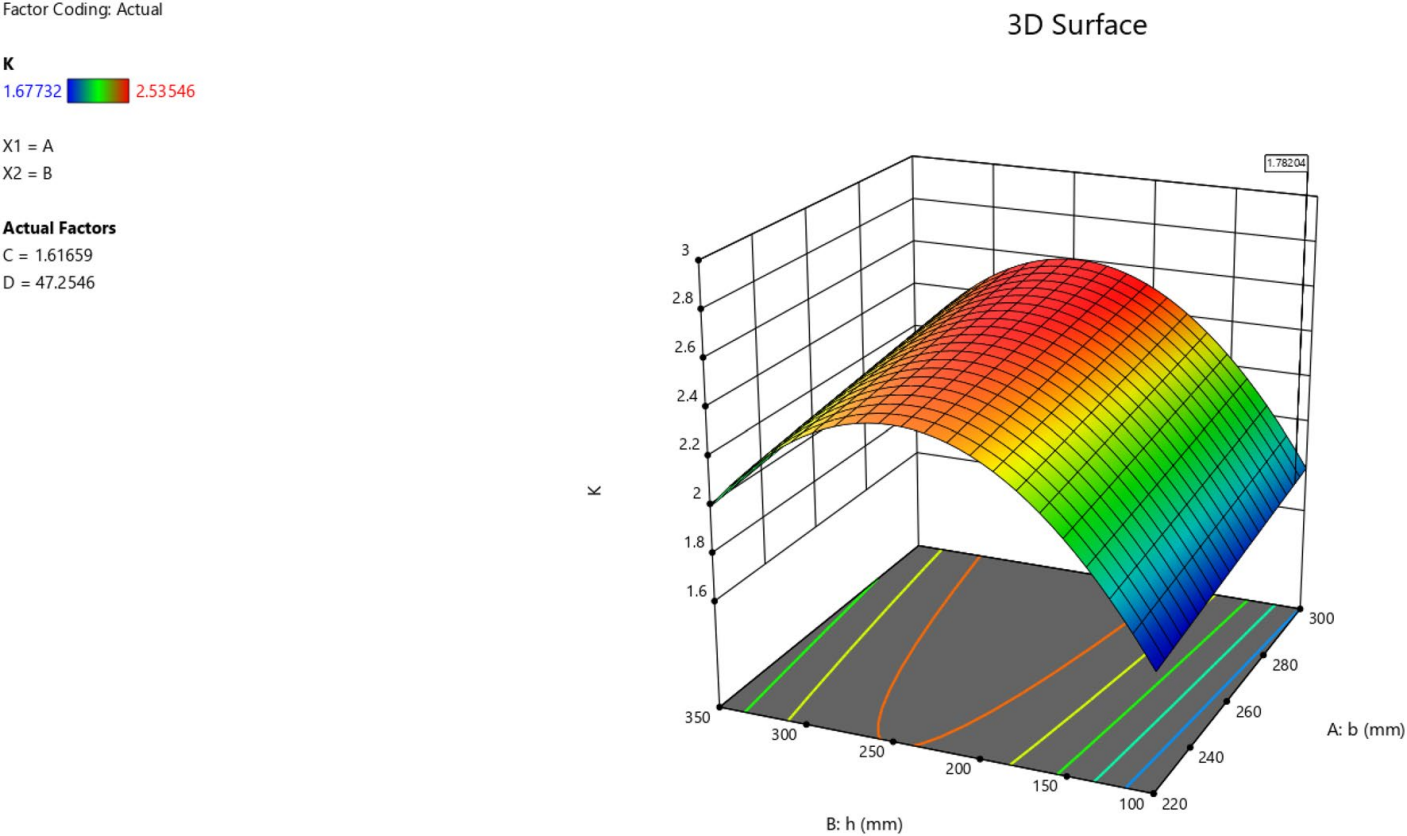
The color coding for the 3D surface of the RSM K minimization model.

The first iteration produced 12 solutions for the maximization of the K model as presented in Figs. 9, 10, 11 and 12 as the desirability level, the factor coding for the perturbation, factor coding and the 3D surface while the second iteration produced 72 solutions for the minimization of the K and the desired solutions were used to validate the model as also presented in Figs. 13, 14, 15 and 16 as the desirability level, the factor coding for the perturbation, factor coding and the 3D surface.

#### Steel damper slenderness factor (λ)

The maximum model order was set to quadratic for process factors, the properties of which are shown in Tables 12, 13 and 14. The selected model on the Model tab may be the design model or lower in order. The fit summary calculation in Tables 12, 13 and 14 was ended prematurely based on options set on the Transform tab. Select the highest order polynomial where the additional terms are significant and the model is not aliased. Focus on the model maximizing the Adjusted R^2^ and the Predicted R^2^.

**Table 12:** The slenderness factor fit summary.

Source	Sequential p-value	Lack of fit p-value	Adjusted R^2^	Predicted R^2^	
Linear	0.0626	0.0614	0.1608	0.0758	Suggested
2FI	0.6111	0.0533	0.1208	− 0.0548	Aliased

**Table 13:** The slenderness factor sequential model Sum of Squares [Type I].

Source	Sum of squares	df	Mean square	F-value	p-value	
Mean vs. total	840.07	1	840.07			
Linear vs. mean	32.31	4	8.08	2.53	0.0626	Suggested
2FI vs. linear	9.12	4	2.28	0.6822	0.6111	Aliased
Residual	80.20	24	3.34			
Total	961.69	33	29.14

**Table 14:** Model summary statistics.

Source	Std. dev	R^2^	Adjusted R^2^	Predicted R^2^	PRESS	
Linear	1.79	0.2657	0.1608	0.0758	112.40	Suggested
2FI	1.83	0.3406	0.1208	− 0.0548	128.29	Aliased

**Figure 17:**
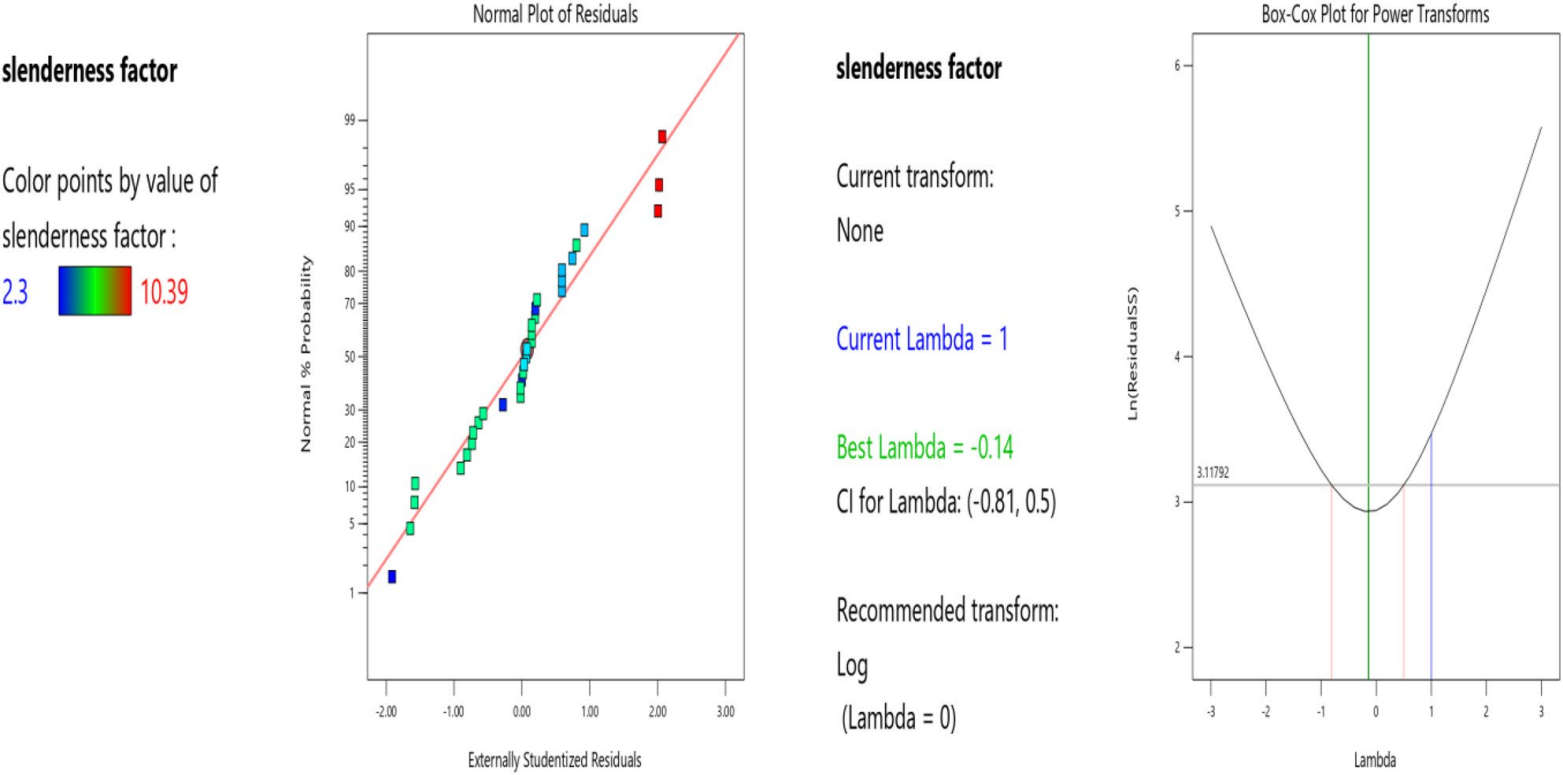
The normal plots of residuals and box-Cox power transform of **λ** model.

**Figure 18:**
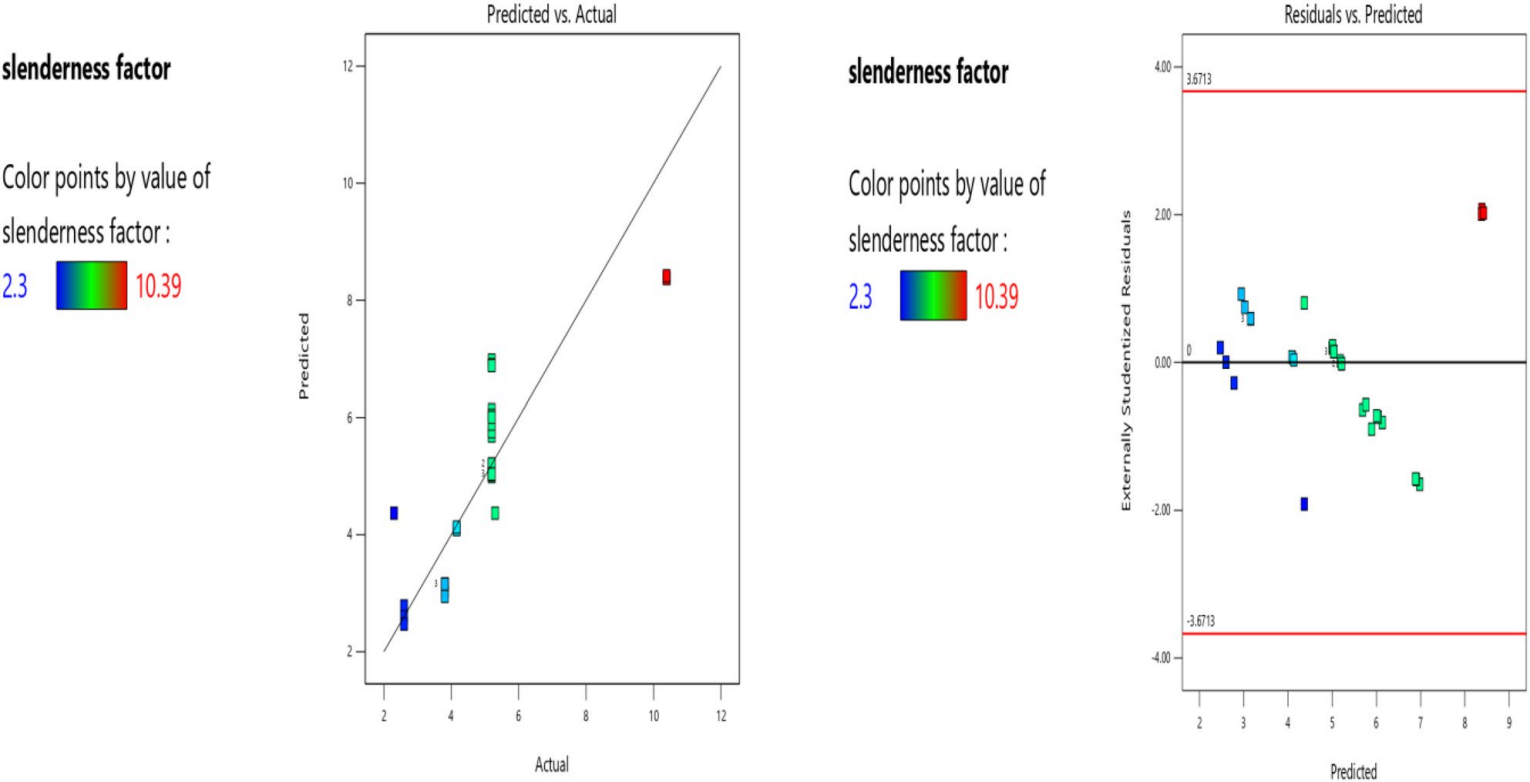
The color points for the predicted/actual and residual/predicted **λ** values.

**Figure 19:**
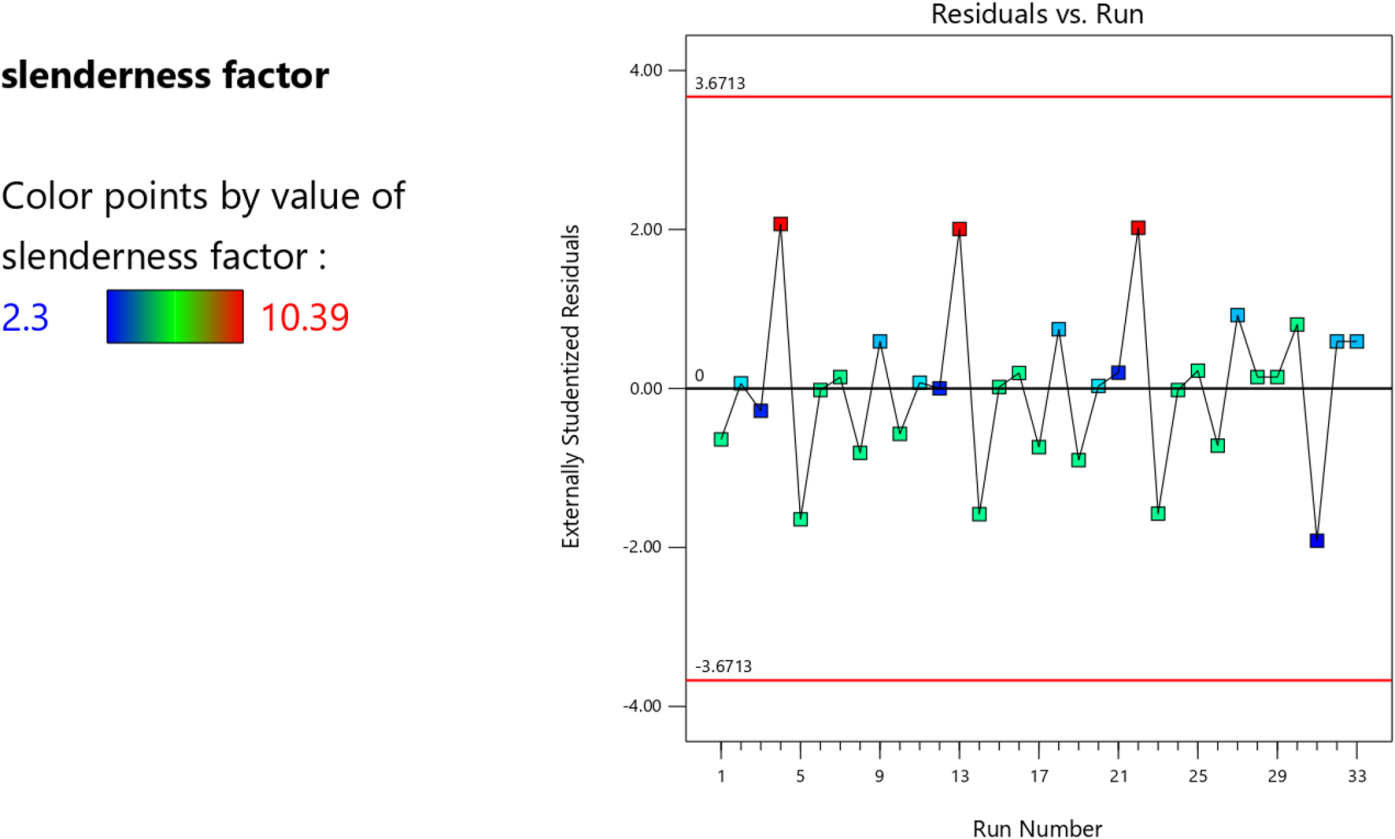
The color points for the residual/run of the RSM **λ** model.

**Figure 20:**
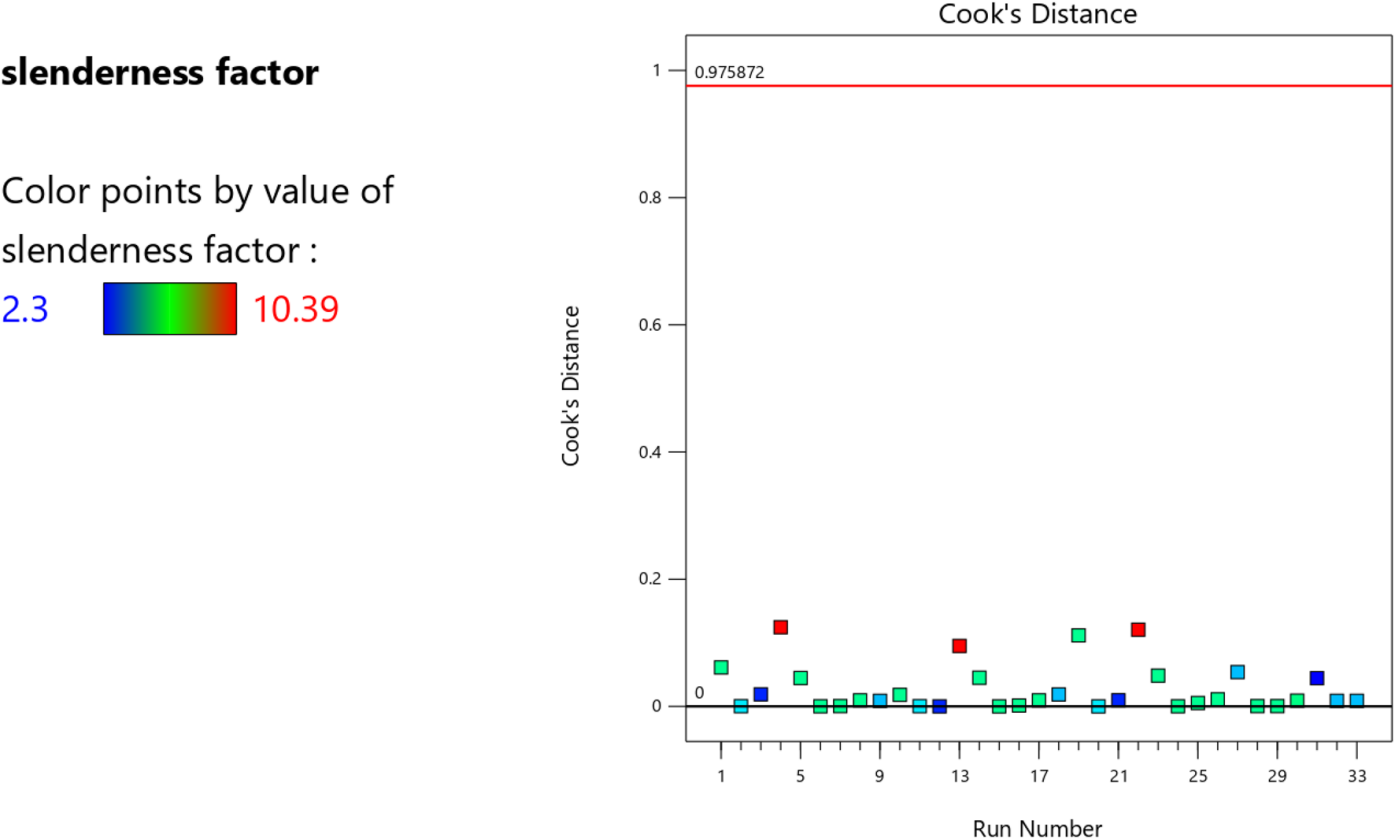
The color points for the Cook’s distance of the RSM **λ** model.

**Figure 21:**
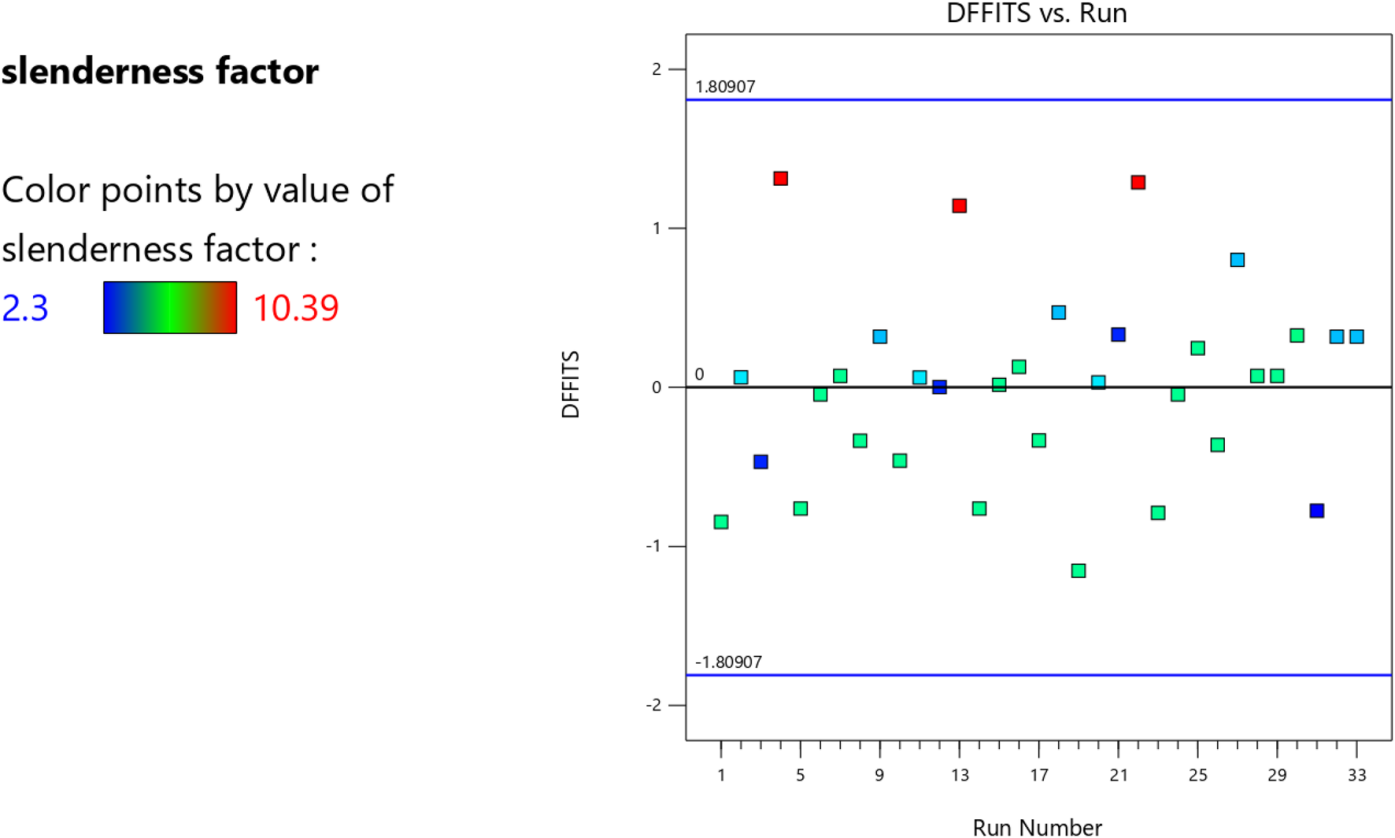
The color points for the degree of fitness of the RSM **λ** model.

Factor coding is Actual**.** Sum of squares is Type III—Partial. The Model F-value of 5.31 implies the model is significant. There is only a 0.05% chance that an F-value this large could occur due to noise. P-values less than 0.0500 indicate model terms are significant. In this case B, BC, B2, C2 are significant model terms. Values greater than 0.1000 indicate the model terms are not significant. If there are many insignificant model terms (not counting those required to support hierarchy), model reduction may improve your model. The Lack of Fit F-value of 1.92 implies the Lack of Fit is not significant relative to the pure error. There is a 24.28% chance that a Lack of Fit F-value this large could occur due to noise. Non-significant lack of fit is good—we want the model to fit. The Predicted R^2^ of 0.5133 is in reasonable agreement with the Adjusted R^2^ of 0.5971; i.e. the difference is less than 0.2. Adeq Precision measures the signal to noise ratio. A ratio greater than 4 is desirable. Your ratio of 7.972 indicates an adequate signal. This model can be used to navigate the design space. The coefficient estimate represents the expected change in response per unit change in factor value when all remaining factors are held constant. The intercept in an orthogonal design is the overall average response of all the runs. The coefficients are adjustments around that average based on the factor settings. When the factors are orthogonal the VIFs are 1; VIFs greater than 1 indicate multi-colinearity, the higher the VIF the more severe the correlation of factors. As a rough rule, VIFs less than 10 are tolerable. The deployed model properties are presented in Tables 15, 16, 17. The Eq. (12) in terms of actual factors can be used to make predictions about the response for given levels of each factor. Here, the levels should be specified in the original units for each factor. This equation should not be used to determine the relative impact of each factor because the coefficients are scaled to accommodate the units of each factor and the intercept is not at the center of the design space. Figures 17, 18, 19, 20 and 21 presents the ANOVA-RSM interface which modeled and optimizes the slenderness factor (**λ**) of the steel damper.

**Table 15:** The slenderness factor ANOVA for Quadratic model (Aliased).

Source	Sum of squares	df	Mean square	F-value	p-value	
Model	89.46	11	8.13	5.31	0.0005	Significant
A-b (mm)	3.59	1	3.59	2.35	0.1404	
B-h (mm)	38.72	1	38.72	25.28	< 0.0001	
C-tp (mm)	3.44	1	3.44	2.25	0.1487	
D-tb (mm)	0.0001	1	0.0001	0.0001	0.9925	
AB	0.0000	0				
AC	0.0000	0				
AD	0.0030	1	0.0030	0.0019	0.9653	
BC	12.40	1	12.40	8.10	0.0097	
BD	0.0762	1	0.0762	0.0497	0.8257	
CD	0.0726	1	0.0726	0.0474	0.8297	
A^2^	0.0000	0				
B^2^	41.55	1	41.55	27.13	< 0.0001	
C^2^	9.16	1	9.16	5.98	0.0234	
D^2^	0.0048	1	0.0048	0.0031	0.9559	
Residual	32.16	21	1.53			
Lack of fit	27.66	16	1.73	1.92	0.2428	Not significant
Pure error	4.50	5	0.9000			
Cor total	121.63	32

**Table 16:** Fit Statistics.

Std. dev	1.24	R^2^	0.7356
Mean	5.05	Adjusted R^2^	0.5971
C.V. %	24.53	Predicted R^2^	0.5133
		Adeq precision	7.9716

**Table 17:** Coefficients in Terms of Actual Factors.

Factor	Coefficient estimate	df	Standard error	95% CI low	95% CI high	VIF
Intercept	− 31.62	1	17.31	− 67.62	4.38	
A-b (mm)	0.0832	1	0.0543	− 0.0297	0.1961	33.60
B-h (mm)	0.2050	1	0.0408	0.1202	0.2898	263.39
C-tp (mm)	− 3.83	1	2.55	− 9.14	1.48	93.30
D-tb (mm)	− 0.0050	1	0.5271	− 1.10	1.09	415.67
AB	Aliased					
AC	Aliased					
AD	− 0.0001	1	0.0013	− 0.0027	0.0026	245.52
BC	− 0.0398	1	0.0140	− 0.0688	− 0.0107	370.18
BD	− 0.0001	1	0.0004	− 0.0009	0.0007	39.97
CD	0.0092	1	0.0421	− 0.0784	0.0968	48.96
A^2^	Aliased					
B^2^	− 0.0002	1	0.0000	− 0.0003	− 0.0001	64.74
C^2^	1.82	1	0.7453	0.2727	3.37	191.01
D^2^	0.0003	1	0.0050	− 0.0100	0.0106	240.85

12 $${\varvec{\uplambda}}= -0.00247{h}^{2}+1.82259{tp}^{2}+0.000278{tb}^{2}+0.083189 b+0.205034 h-3.82986 tp-0.005041 tb-0.000056 b*tb-0.039770h*tp-0.000083h*tb+0.009174 tb*tp-31.62048$$

**Figure 22:**
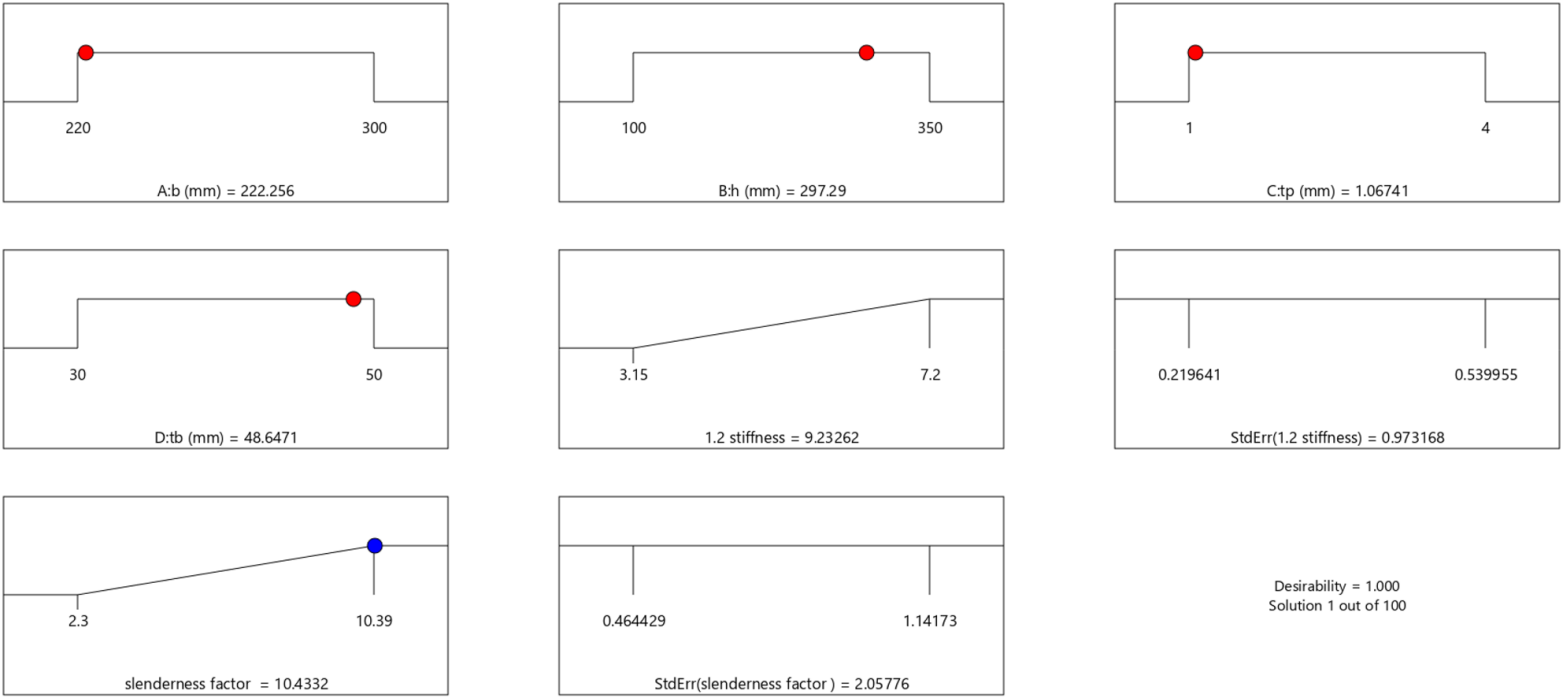
The desirability level of the RSM **λ** maximization model.

**Figure 23:**
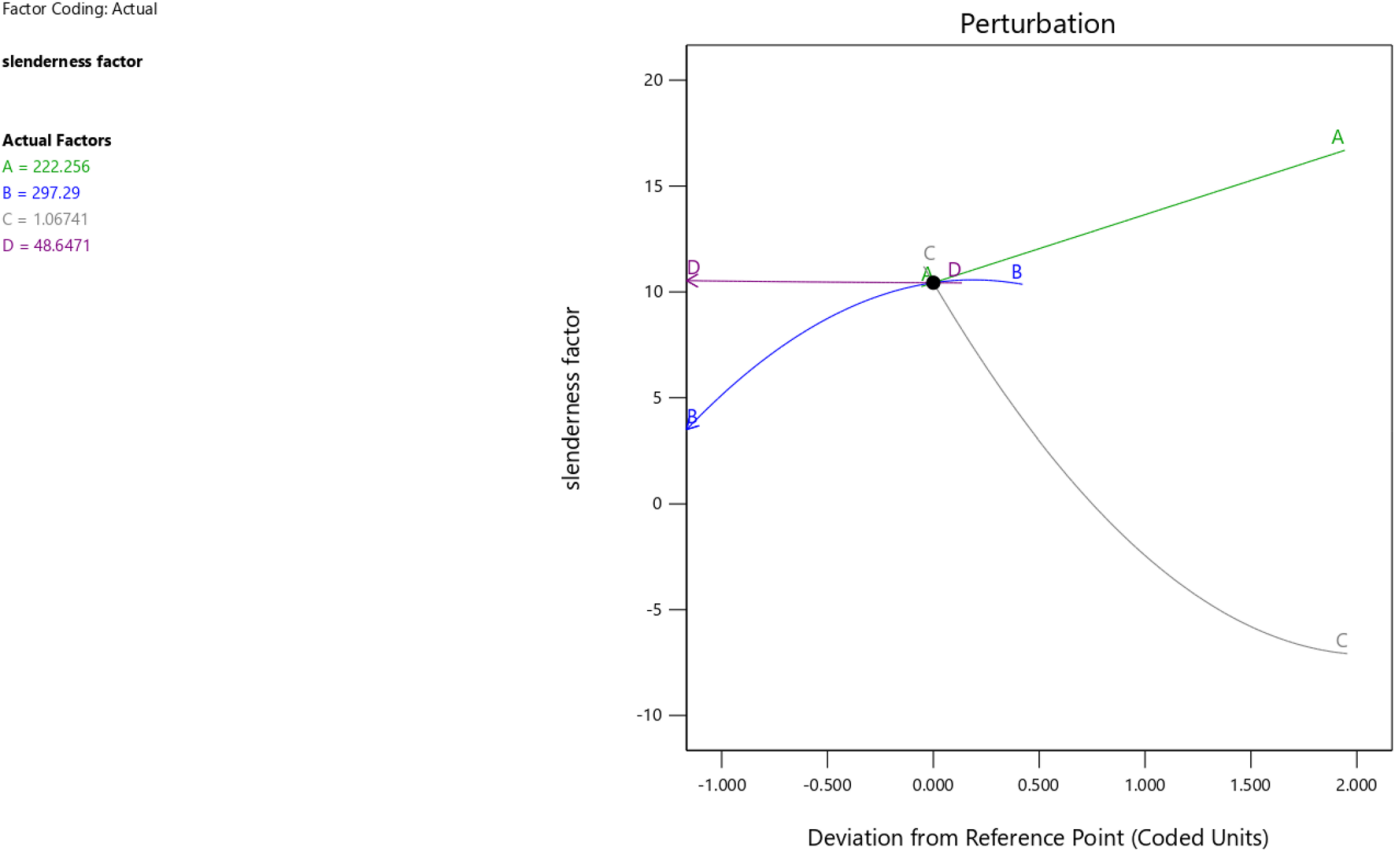
The factor coding for the perturbation of the RSM **λ** maximization model.

**Figure 24:**
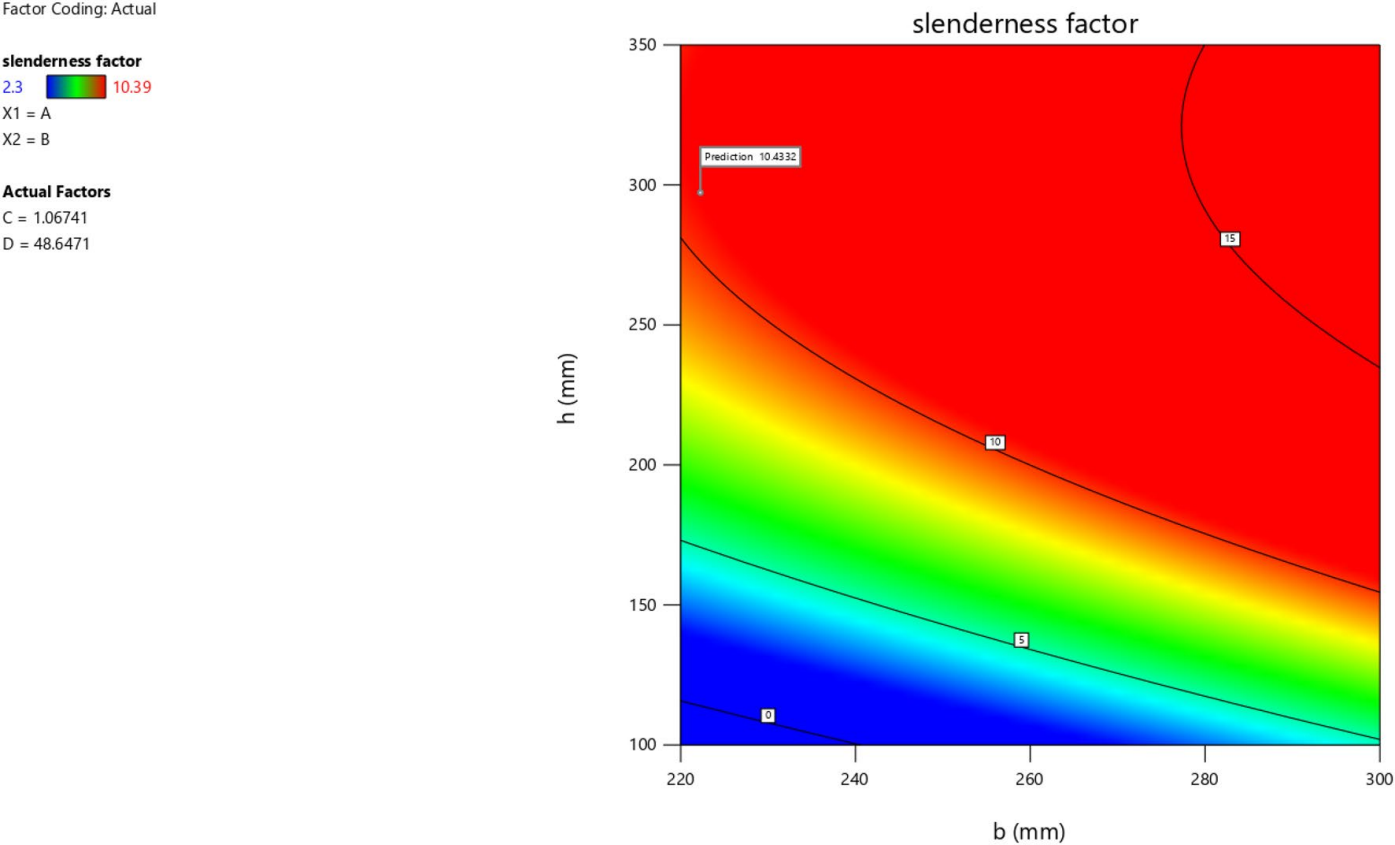
The color coding for the factor coding of the RSM **λ** maximization model.

**Figure 25:**
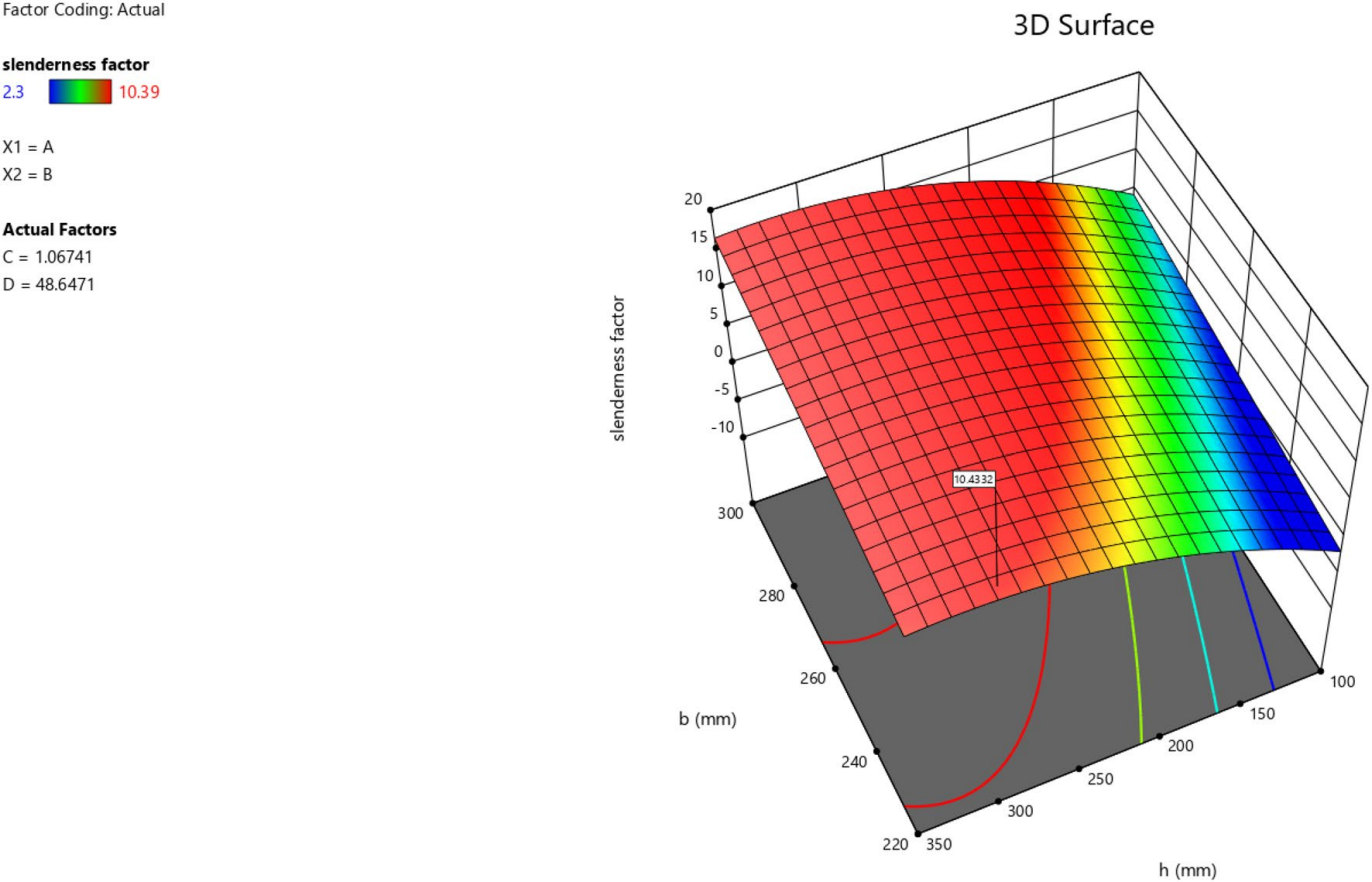
The color coding for the 3D surface of the RSM **λ** maximization model.

**Figure 26:**
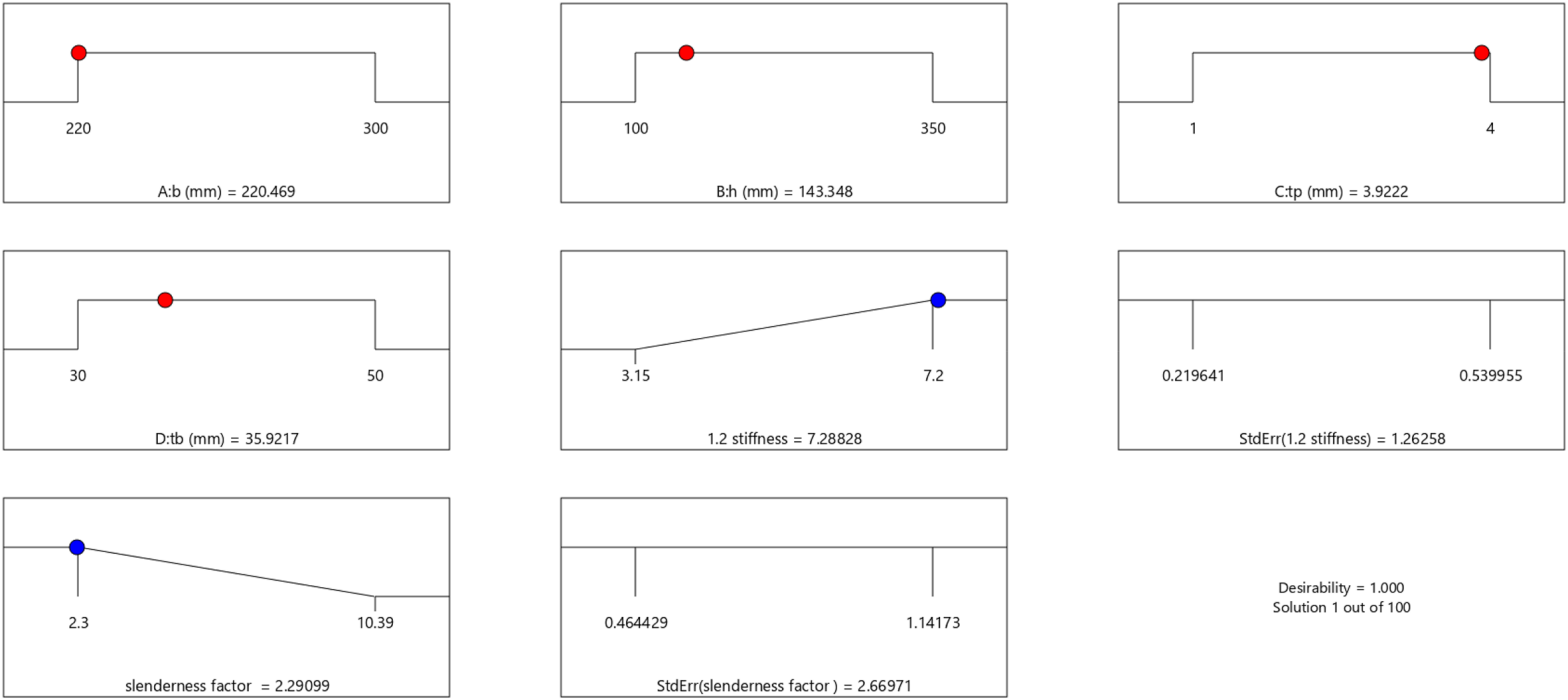
The desirability level of the RSM **λ** minimization model.

**Figure 27:**
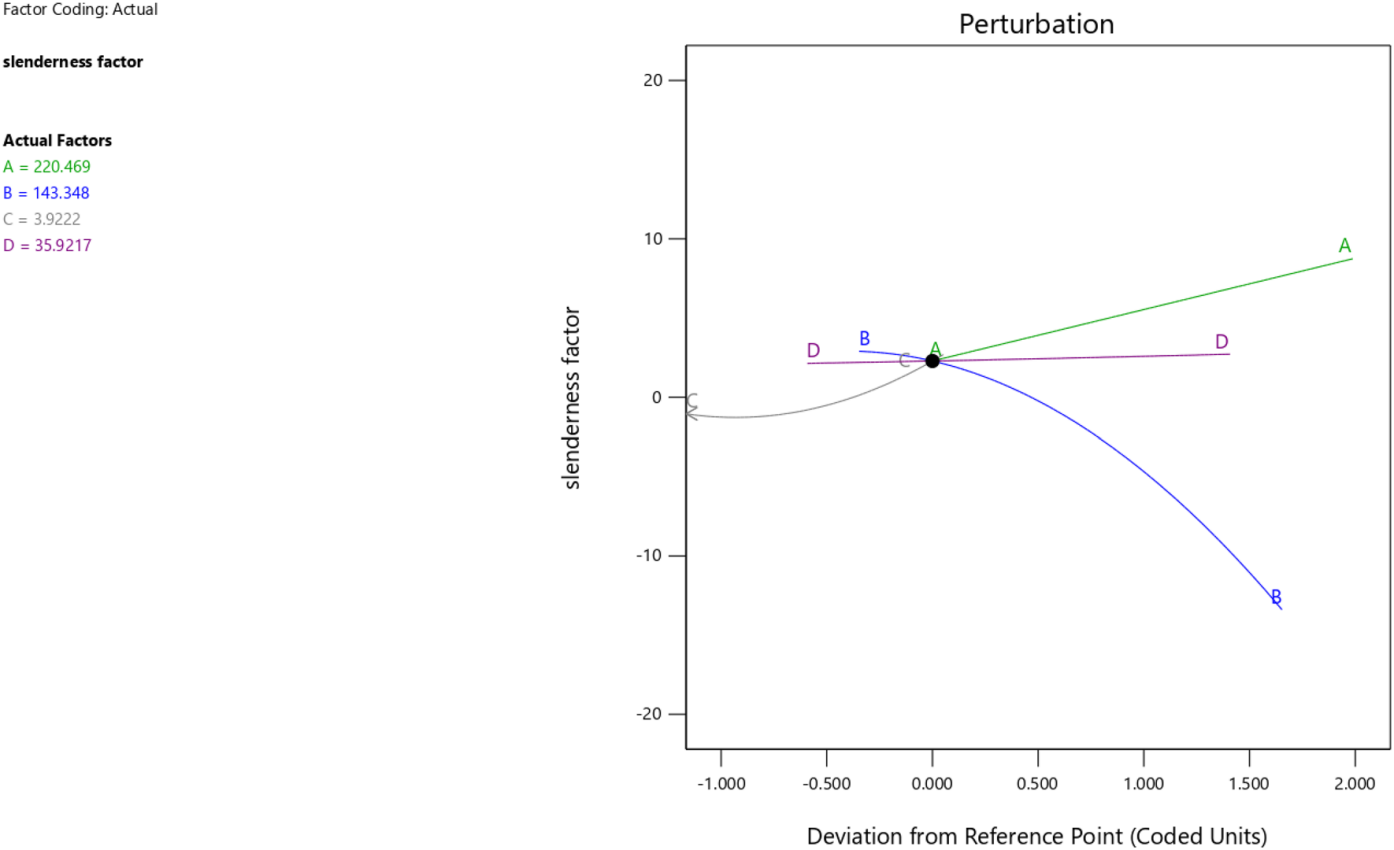
The factor coding for the perturbation of the RSM **λ** minimization model.

**Figure 28:**
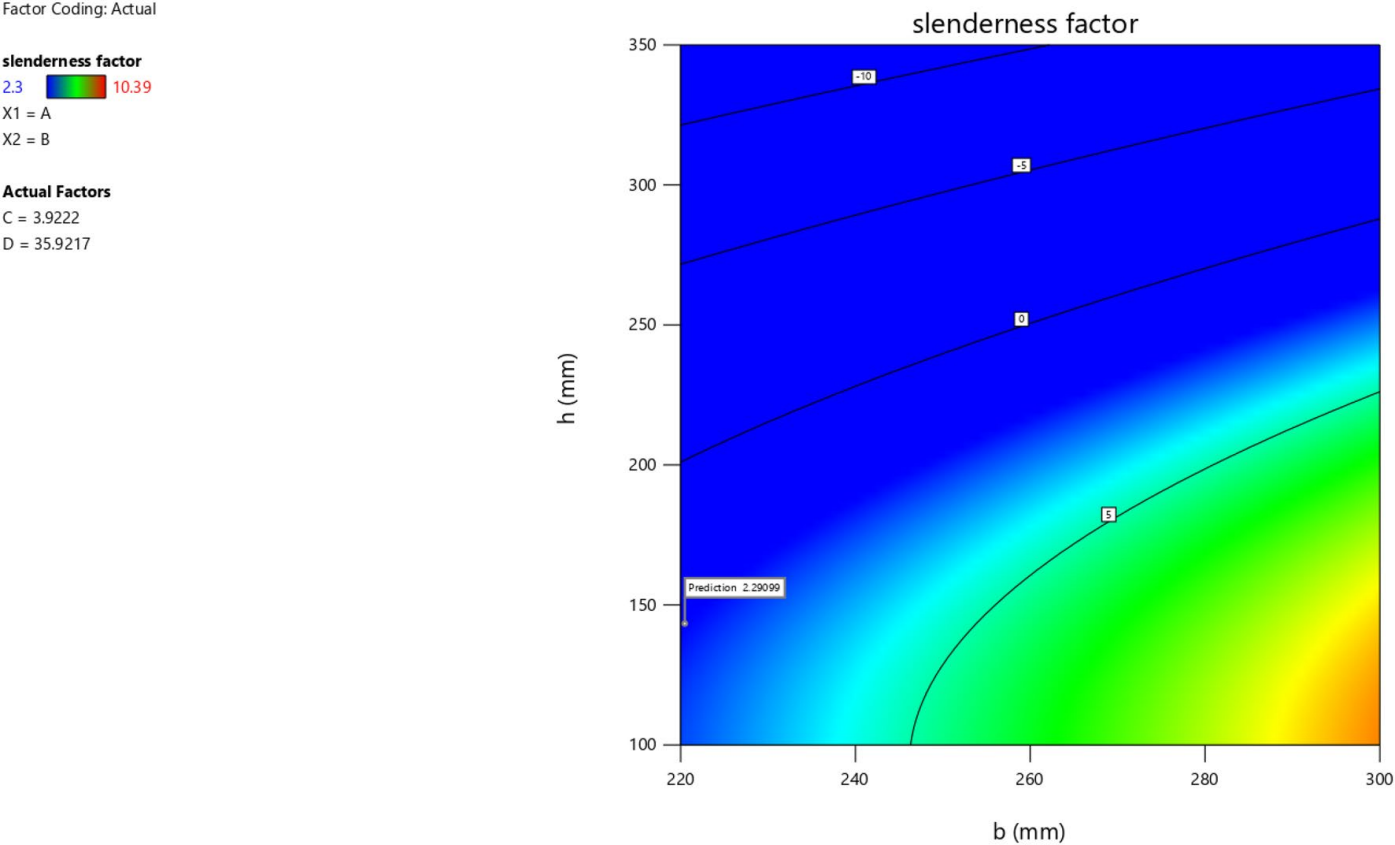
The color coding for the factor coding of the RSM **λ** minimization model.

**Figure 29:**
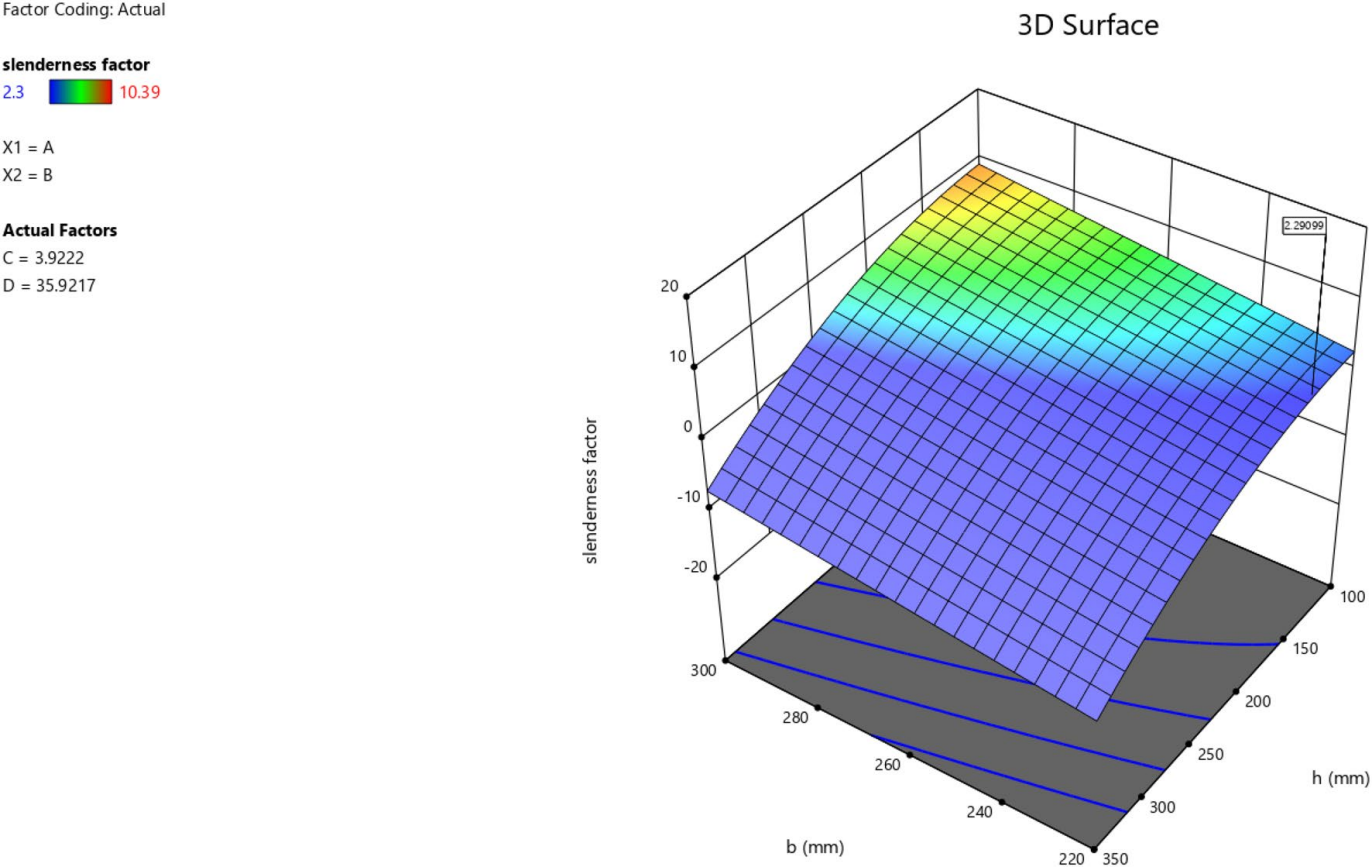
The color coding for the 3D surface of the RSM **λ** minimization model.

In the maximization of the model, 100 solutions were found in the maximization iteration and solution 1 was selected and in Fig. 22, 23, 24 and 25 are presented the desirability level, the factor coding for the perturbation, factor coding and the 3D surface while 100 solutions were found in the minimization iteration and solution 1 was also selected and in Fig. 26, 27, 28 and 29 are also presented the desirability level, the factor coding for the perturbation, factor coding and the 3D surface. Overall, Table 18 shows the performance evaluation summary of the RSM models, which produced MAE of 0.31695, MSE of 0.60659, RMSE of 0.77884 and R^2^ of 0.7356 for the steel plate damper slenderness factor (λ) and MAE of 0.0232, MSE of 0.00297, RMSE of 0.054473 and R^2^ of 0.8925 for the steel plate damper stiffness (K). Furthermore, some advanced machine learning techniques have been deployed to improve the intelligent performance of the studied characteristics of the steel plate damper for a more sustainable technological advanced prediction, design and construction. Overall, previous research papers have presented the behavior mechanism of various damper systems^29–31,35^, but none presented intelligent models like it is done in this present research paper.

**Table 18:** RSM performance summary.

	1.12 root (k)	Slenderness factor	1.4 root (k)	K
RMSE	0.15938	0.77884	0.10883	0.054473
R-Squared	0.8822	0.7356	0.882	0.8925
MSE	0.025401	0.60659	0.011845	0.0029673
MAE	0.11067	0.31695	0.045046	0.023173

## Solutions

### ANN model analysis

**Figure 30:**
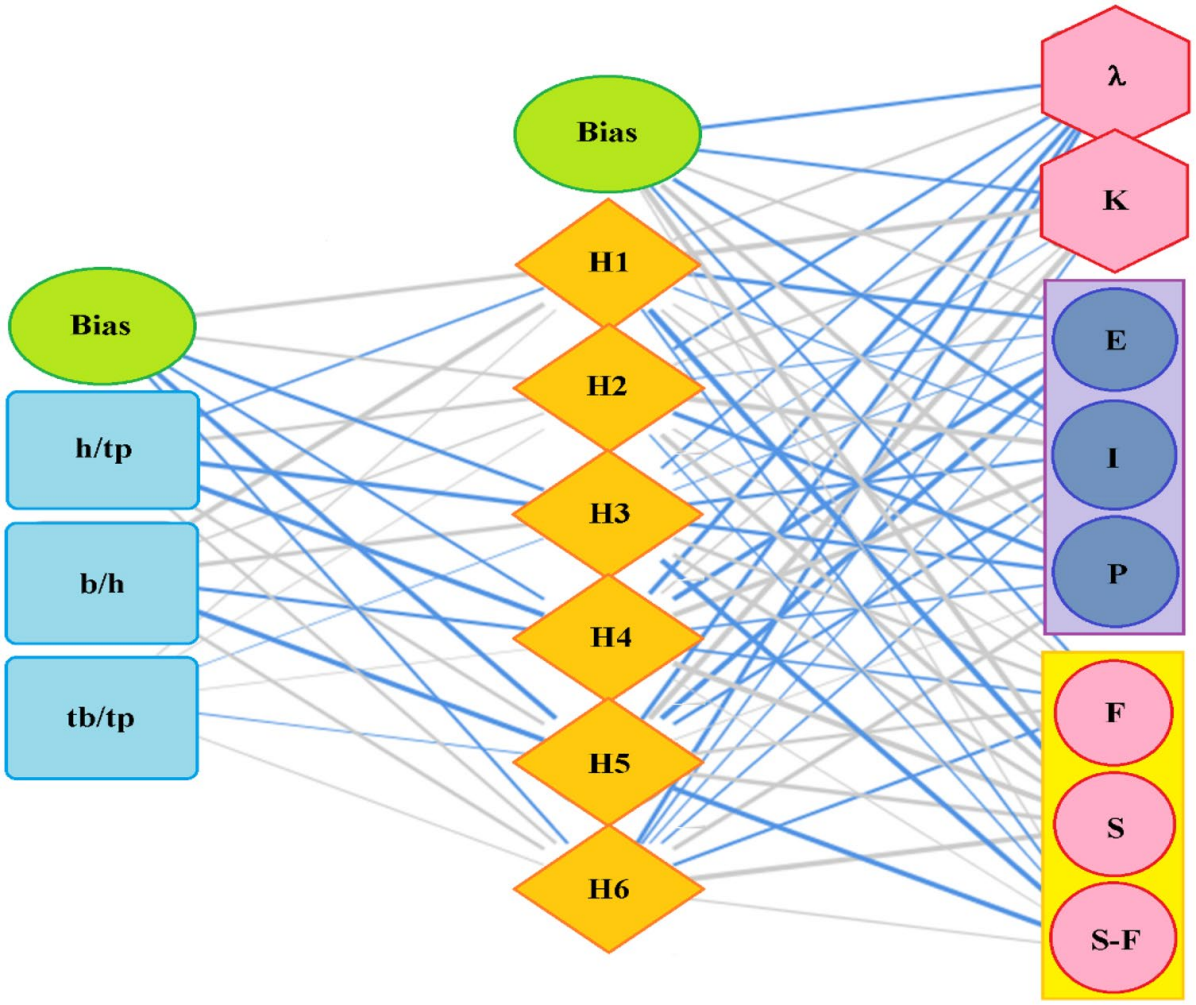
The architecture of the ANN model.

**Figure 31:**
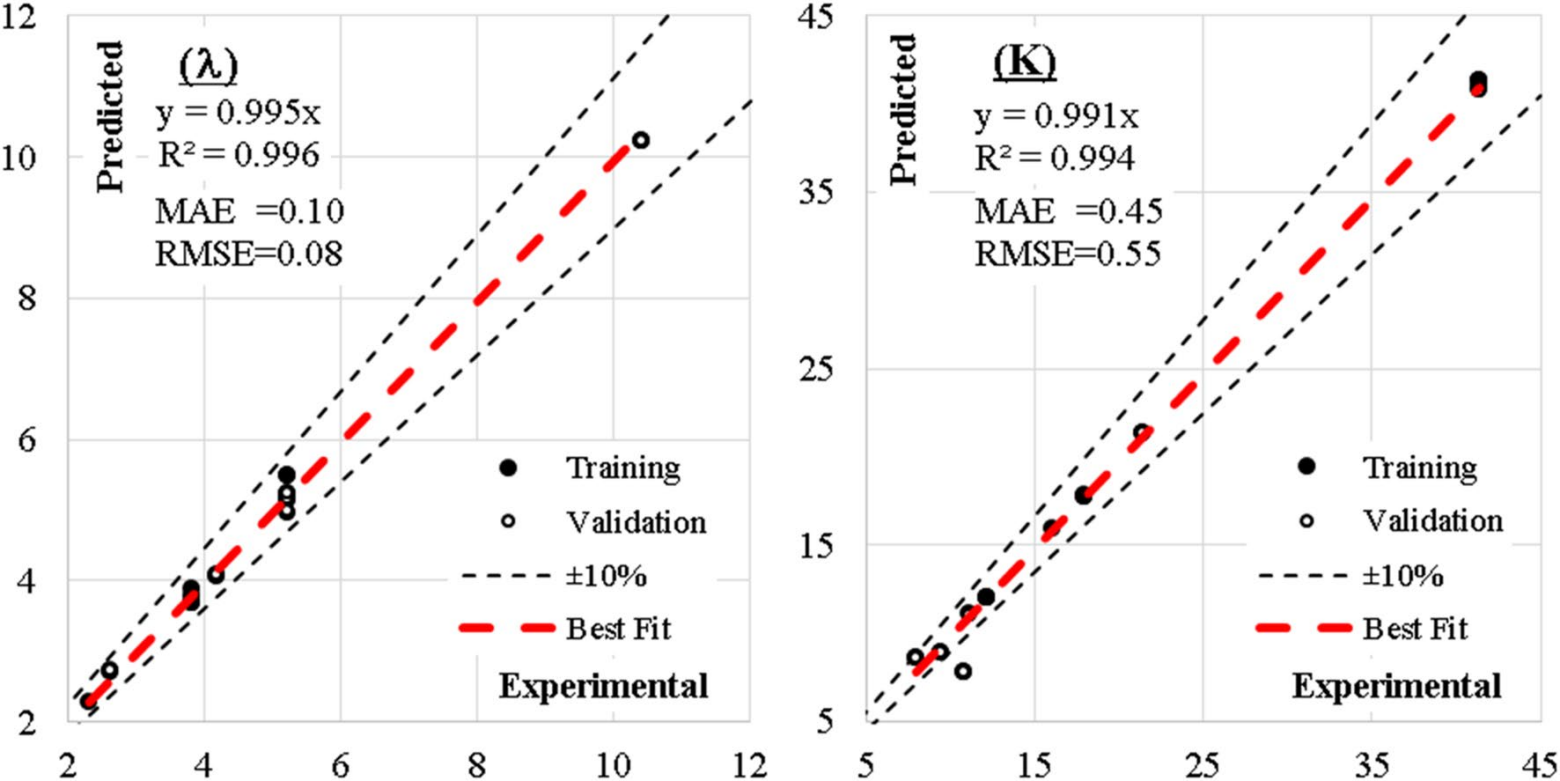
The best line of fit between measured and predicted values based on ANN model.

**Figure 32:**
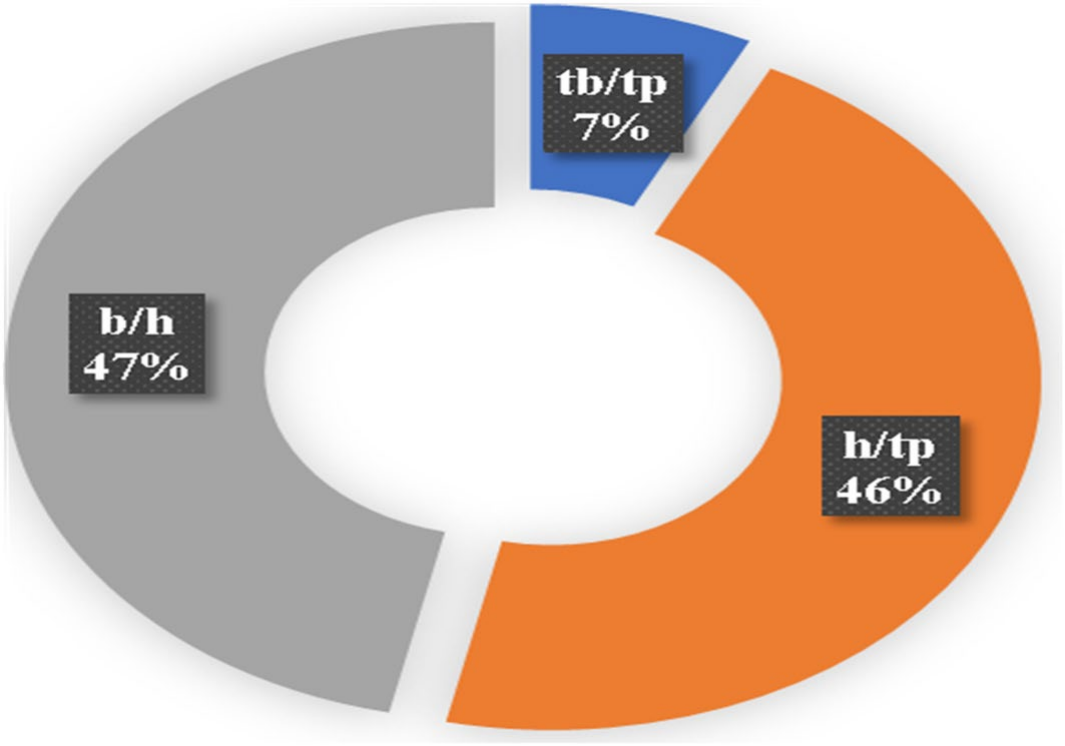
The degree of importance of the parameters on the damper slenderness factor and stiffness.

A 3-6-8 ANN topology as shown in Fig. 30 and the weight and biases presented in Table 19 have been used in this unique model, which studied both the fundamental output of a steel damper and the damper’s mode and mechanics of failure. The hyper-tahn activation function and the popular back-propagation training algorithm were used in the ANN interface to model damper slenderness factor (λ), stiffness (K) and the mode (EIP) and the mechanism (FSS-F) of failure upon loading. The comparison between the measured and predicted values of the steel damper as presented in Table 20 and Fig. 31 produced R^2^ of 0.996 and 0.994, respectively for λ and K. The further evaluated performance errors of the ANN models show the SSE, MAE, MSE, and RMSE produced 0.02, 0.10, 0.01, and 0.08 for $\uplambda$ and 0.03, 0.45, 0.31, and 0.55 for K, respectively. The degree of importance of the parameters on the damper slenderness factor ($\uplambda$) and stiffness (K) based on the ANN interface presented in Fig. 32 produced b/h ratio as the most important ratio factor with a significance of 47%, which is closely followed by b/tp ratio with a significance of 46%, and this unarguably agrees with^10^. These show that these two ratios (b/h and b/tp) are to be considered seriously in the design of steel plate dampers for the efficient performance of steel structures. Previous research papers have presented the behavior mechanism of various damper systems^29–31,35^, but none presented intelligent models like it is done in this present research paper.

**Table 19:** The weight and biases of the ANN model.

	H (1:1)	H (1:2)	H (1:3)	H (1:4)	H (1:5)	H (1:6)	
(Bias)	8.03	1.55	− 3.26	− 1.42	− 8.98	− 0.90	
h/tp	− 0.65	2.74	− 9.65	− 13.60	2.07	1.80	
b/h	12.25	0.40	4.73	− 2.37	− 13.66	2.10	
tb/tp	0.04	0.01	− 0.01	0.01	− 0.02	0.06	

	H(1:1)	H(1:2)	H(1:3)	H(1:4)	H(1:5)	H(1:6)	(Bias)
λ	0.80	− 1.86	− 0.76	− 4.66	− 3.40	− 3.96	− 2.97
K	18.21	0.64	− 0.02	0.34	17.49	− 0.42	− 1.83
E	− 4.64	− 0.05	− 0.02	− 10.29	− 13.71	− 0.07	1.17
I	− 0.16	17.00	− 1.93	14.30	− 1.93	− 0.54	− 3.30
P	0.22	− 8.53	− 3.91	− 0.61	0.01	4.26	7.80
F	1.04	0.57	3.00	− 0.77	2.02	− 1.83	− 0.89
S	− 16.56	9.69	4.09	19.01	7.57	15.10	16.08
S-F	− 0.59	− 0.08	− 12.45	0.02	− 12.94	0.10	0.08

**Table 20:**
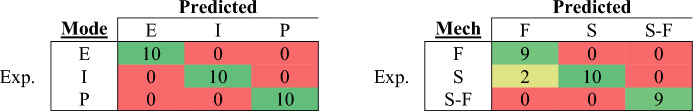
The comparison between measured and predicted values of the mode and mechanics of failure based on the ANN model.

### EPR model analysis

**Figure 33:**
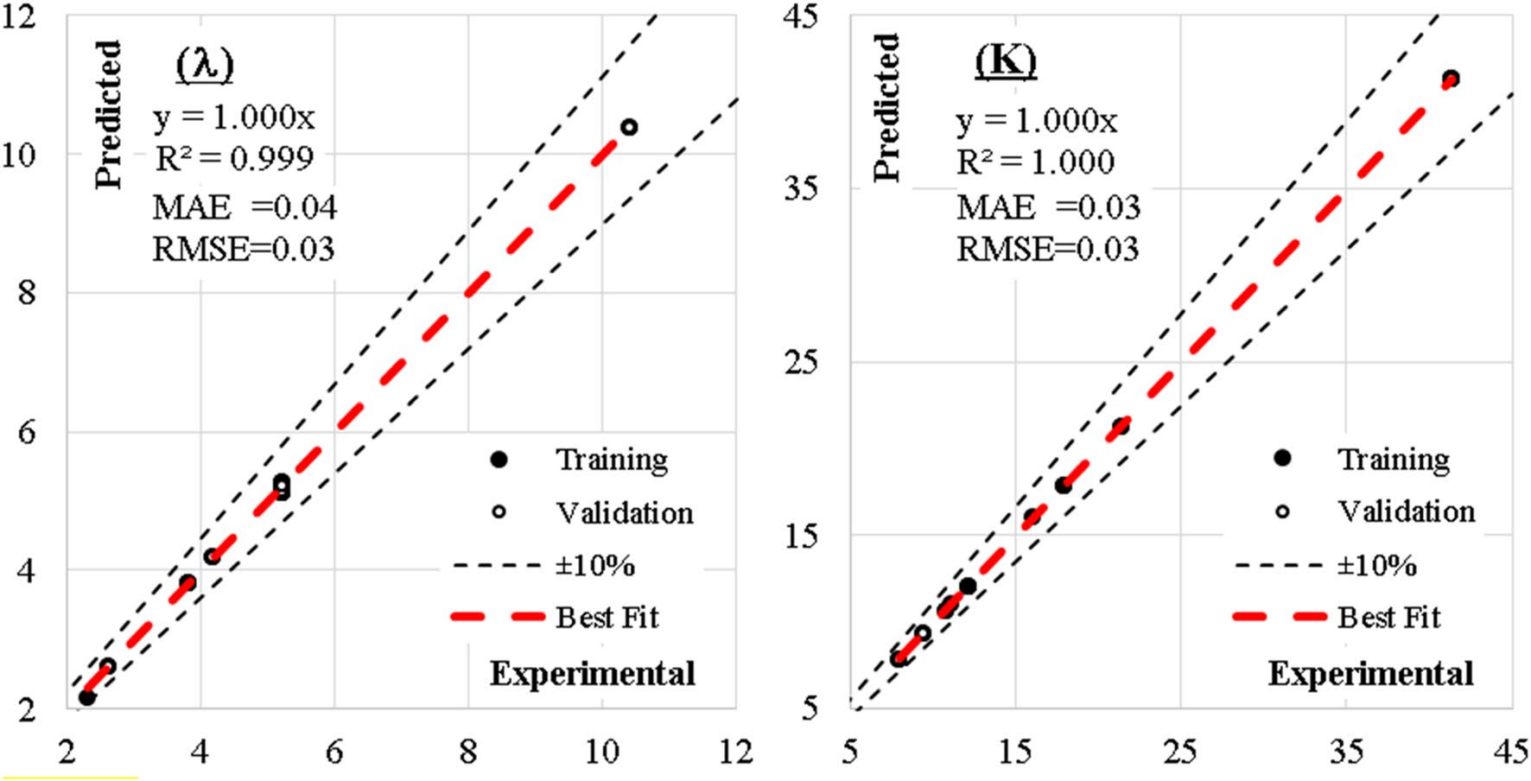
The best line of fit between measured and predicted values based on EPR model.

**Figure 34:**
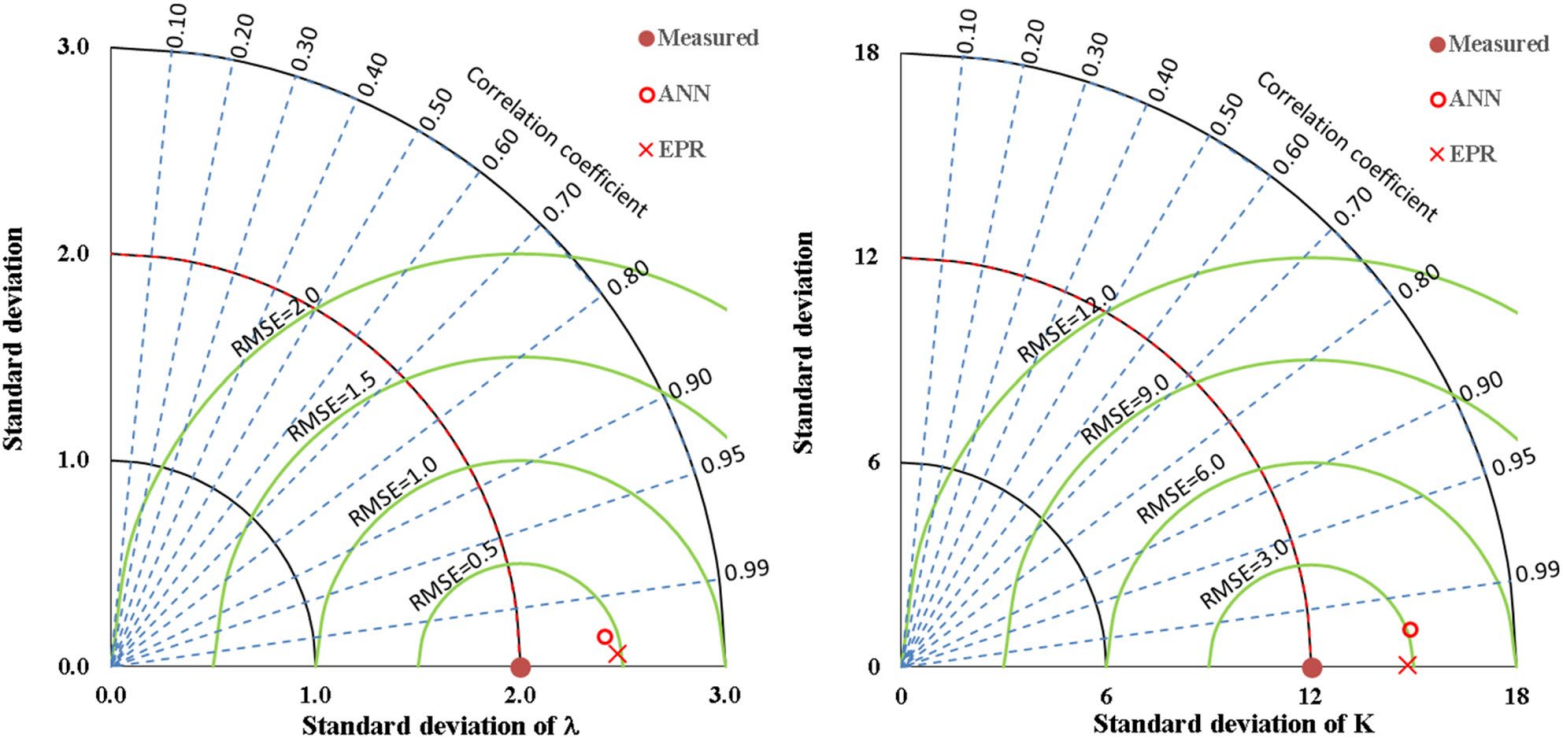
The Taylor chart comparing the performance of the models based on RMSE and R^2^.

The mode of the damper failure closed-form equation is valid within the following ranges; Mode ≤ 0.66; Elastic buckling, 0.66 < Mode ≤ 1.33; Inelastic buckling, Mode > 1.33; Plastic buckling. Also, the mechanism of damper failure closed-form equation is valid within the following ranges; Mech ≤ 0.66; Flexural failure, 0.66 < Mech ≤ 1.33; Shear failure, Mech > 1.33; Flexural-Shear failure. The predicted and measured values have been compared using the EPR model interface as presented in Table 21 and Fig. 33. The slenderness factor ($\uplambda$) and damper stiffness (K) compared well with the measured and predicted values with R^2^ of 0.999 and 1.000, respectively. This performance has been illustrated in the closed-form Eqs. (13) and (14). The Eqs. (15) and (16) present the mode and mechanics of failure for the damper. The evaluated performance of the EPR models show the SSE, MAE, MSE, and RMSE produced 0.08, 0.04, 0.001, and 0.03 for $\uplambda$ and 0.08, 0.03, 0.001, and 0.003 for K, respectively. Table 22 presents the summary of the overall performance metrics while Fig. 34 further reinforced the performance check metrics of the RMSE and R-squared using the Taylor chart. Previous research papers have presented the behavior mechanism of various damper systems^29–31,35^, but none presented intelligent models like it is done in this present research paper.

**Table 21:**
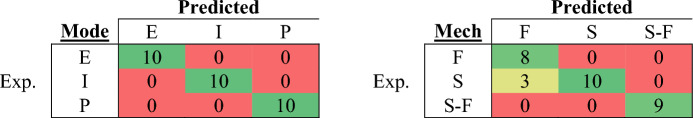
Comparison between measured and predicted failure mode and mechanics.

**Table 22:** Summary of the models’ performance evaluation.

Model	Output	SSE	MAE	MSE	RMSE	Error %	R^2^
ANN	λ	0.02	0.10	0.01	0.08	1.5	0.996
	K	0.03	0.45	0.31	0.55	3.0	0.994
	Mode	–	–	–	–	0.0	–
	Mech	–	–	–	–	6.7	–
EPR	λ	0.08	0.04	0.001	0.03	0.6	0.999
	K	0.08	0.03	0.001	0.03	0.2	1.000
	Mode	–	–	–	–	0.0	–
	Mech	–	–	–	–	10.0	–

13 $$\uplambda =\frac{1.3\times {10}^{7}{{\text{tp}}}^{3}}{{{\text{h}}}^{3}} +\frac{11.4 {{\text{b}}}^{2}}{{{\text{h}}}^{2}}+\frac{304\mathrm{ h}}{{\text{b}}}-\frac{{{\text{h}}}^{2}}{2.5\mathrm{ tp}.{\text{b}}}-\frac{12730\mathrm{ tp}.{{\text{h}}}^{2}}{{{\text{b}}}^{3}}-\frac{34745\mathrm{ t}{{\text{p}}}^{2}.{\text{b}}}{{{\text{h}}}^{4}}-162$$

14 $${\text{K}}=\frac{1.13\mathrm{ h}}{{\text{tp}}}-\frac{2.3\mathrm{ b}}{{\text{tp}}}+\frac{0.55\mathrm{ b}}{{\text{tp}}.{\text{h}}}+\frac{1.66 {{\text{h}}}^{3}}{{\text{tp}}.{{\text{b}}}^{2}}-\frac{387\mathrm{ tp}.{{\text{b}}}^{2}}{{{\text{h}}}^{3}}-\frac{230 {{\text{h}}}^{4}}{{{\text{b}}}^{4}}+120.5$$

15 $${\text{Mode}}= \frac{{{\text{h}}}^{2}}{{372\mathrm{ b}.{\text{tp}}}^{2}}-\frac{103400 {{\text{tp}}}^{2}}{{{\text{b}}}^{2}}+\frac{63.4 {{\text{h}}}^{2}}{{{\text{b}}}^{2}}-\frac{0.48{\mathrm{ h}}^{2}}{{\text{tp}}.{\text{b}}}-\frac{{0.36\mathrm{ h}}^{3}}{{\text{tp}}.{{\text{b}}}^{2}}-\frac{{{\text{b}}}^{2}}{33310 {{\text{tp}}}^{2}}+8$$

16 $${\text{Mech}}=\frac{31.8 {{\text{b}}}^{2}}{{{\text{h}}}^{2}}-\frac{522.7\mathrm{ h}}{{\text{b}}}+\frac{193.1 {{\text{h}}}^{2}}{{{\text{b}}}^{2}}-\frac{{{\text{h}}}^{3}}{20\mathrm{ tp}.{{\text{b}}}^{2}}-\frac{527\mathrm{ tp}.{\text{h}}}{{{\text{b}}}^{2}}-\frac{220.6\mathrm{ b}}{{\text{h}}}+529.66$$

## Conclusions

The extensive prediction and analyses of a steel plate-based damper properties (λ and K) designed for the improvement of the behavior of concentrically braced frames based on baseline regressions, analysis of variance, response surface methodology optimization and machine learning approach for sustainable structures have been presented in this research paper. The following are the concluding remarks extracted from the results of the general exercise;

- A total of thirty-three data entries were collected from the behavior of the steel plate damper stiffness (K) and slenderness factor (λ) corresponding to the geometrical characteristics (b, h, tb and tp), which were deployed as input parameters.
- The general database was sorted and analyzed using statistical tools, which produced the linear closed-form equations relevant to the statistical metrics
- The database was divided into training and validation sets considering important ratios of the steel plate damper properties and deployed for analyses using the response surface methodology (RSM) minimization and maximization techniques, artificial neural network (ANN) and the evolutionary polynomial regression (EPR).
- The RSM with an incorporated ANOVA interface produced closed-form equations for the outputs (λ and K) in addition to desirability level and color factor optimization scales, which showed the behavior of the output in response to the studied variables.
- The ANN and the EPR produced better models for the λ and K deploying the more robust algorithms than the linear regressions and the RSM-ANOVA.
- Overall, the EPR outclassed all the models and becomes the decisive intelligent model for the prediction of λ and K within the range of value entries considering the mode (elastic/inelastic/plastic buckling; EIP) and mechanics (flexural/shear/flexural-shear; FSS-F) of the failure studied in this project.

## Limitation and recommendation

The research work faced the limitation of limited database related to the steel plate-based damper, which would have caused the over-fitting of the models but this was checked by monitoring the partitioning of the collected database prior to the application of the model techniques. It is recommended that future research activities on this subjected explore more literature to be able to update the current database with new ones.

## Data Availability

The datasets generated and analyzed during the current study are available from the corresponding author on reasonable request.
